# Kinetic modeling of leucine-mediated signaling and protein metabolism in human skeletal muscle

**DOI:** 10.1016/j.isci.2023.108634

**Published:** 2023-12-05

**Authors:** Taylor J. McColl, David C. Clarke

**Affiliations:** 1 Department of Biomedical Physiology and KinesiologySimon Fraser University, Burnaby, BC V5A 1S6, Canada; 2 Centre for Cell Biology, Development and Disease, Simon Fraser University, Burnaby, BC V5A 1S6, Canada

**Keywords:** Biological sciences, Protein

## Abstract

Skeletal muscle protein levels are governed by the relative rates of muscle protein synthesis (MPS) and breakdown (MPB). The mechanisms controlling these rates are complex, and their integrated behaviors are challenging to study through experiments alone. The purpose of this study was to develop and analyze a kinetic model of leucine-mediated mTOR signaling and protein metabolism in the skeletal muscle of young adults. Our model amalgamates published cellular-level models of the IRS1-PI3K-Akt-mTORC1 signaling system and of skeletal-muscle leucine kinetics with physiological-level models of leucine digestion and transport and insulin dynamics. The model satisfactorily predicts experimental data from diverse leucine feeding protocols. Model analysis revealed that total levels of p70S6K are a primary determinant of MPS, insulin signaling substantially affects muscle net protein balance via its effects on MPB, and p70S6K-mediated feedback of mTORC1 signaling reduces MPS in a dose-dependent manner.

## Highlights

• We developed a kinetic model of muscle protein metabolism following leucine feeding
• It integrates modules of mTOR signaling, leucine kinetics, and insulin signaling
• The model predicts that p70S6K levels are important for controlling MPS
• Insulin signaling and p70S6K-mediated feedback on mTORC1 control protein balance

## Graphical abstract

**Figure:**
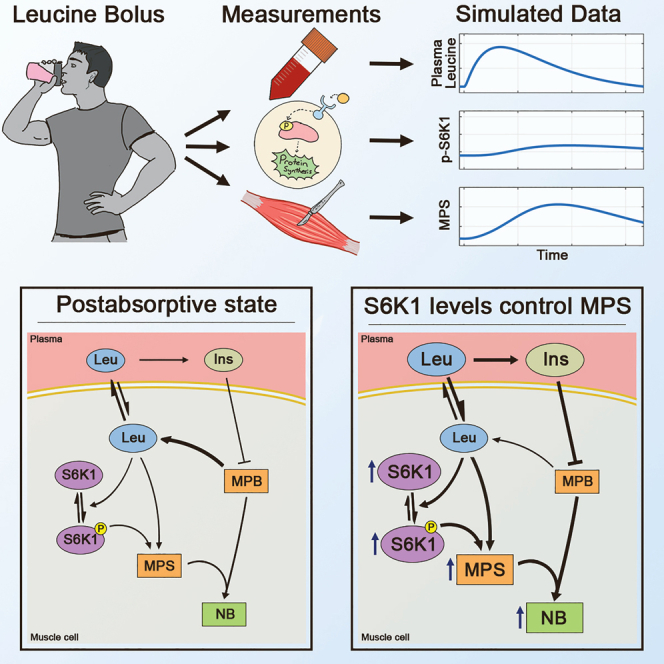

## Introduction

Skeletal muscle enables locomotion, metabolic regulation,^1^ and physical performance.^2^^,^^3^ Skeletal muscle function depends largely on its mass,^4^ such that maintaining or increasing skeletal muscle mass benefits human health and quality of life. Conversely, the loss of skeletal muscle mass in disease states such as sarcopenia negatively impacts health. The prevalence of sarcopenia in older adults (≥60 years) ranges from 9% to 51% depending on factors such as gender, age, pathological condition, and diagnostic criteria.^5^^,^^6^^,^^7^ Those that are sarcopenic are hospitalized more frequently^8^ and engender costlier care.^9^^,^^10^^,^^11^ Therefore, deeper understanding of skeletal muscle mass regulation and effective therapies that promote skeletal muscle mass are sought.

Skeletal muscle mass is determined predominantly by muscle protein levels. Muscle protein levels are governed by the relative rates of muscle protein synthesis (MPS) and muscle protein breakdown (MPB). MPS rates are highly sensitive to amino acid concentrations, in particular leucine,^12^^,^^13^^,^^14^^,^^15^ which promotes protein translation initiation and elongation.^16^^,^^17^^,^^18^ Translation initiation is considered the “rate-limiting step” for protein biosynthesis.^19^ However, feeding only promotes MPS for a finite duration, even with sustained amino acid availability in the blood plasma.^20^^,^^21^ MPB rates vary less than MPS rates.^21^^,^^22^ Thus, the daily fluctuations of MPS above and below MPB, coinciding with fed and post-absorptive states, respectively, are thought to determine overall muscle protein balance. Sustaining a neutral or positive protein balance requires that amino acids be ingested at regular intervals throughout the day.^20^^,^^21^

Muscle protein balance is controlled by three principal mechanisms: 1) hormones (e.g., insulin and insulin-like growth factors, 2) nutrition (e.g., amino acids), and 3) mechanical load (e.g., resistance training).^18^ These factors converge on the protein kinase mechanistic target of rapamycin complex 1 (mTORC1), which integrates these stimuli to control MPS. Of these factors, the amino acid leucine activates mTORC1 through both leucine- and insulin-dependent signaling pathways because leucine also promotes insulin secretion.^23^^,^^24^ Leucine is sensed by Sestrin2, which activates the Ragulator-Rag complex and ultimately activates the mTORC1 kinase.^25^^,^^26^^,^^27^ Insulin activates mTORC1 through a signaling cascade that involves insulin receptor (IR), insulin receptor substrate 1 (IRS1), phosphoinositide 3-kinase (PI3K), 3-phosphoinositide-dependent protein kinase-1 (PDK1), protein kinase B (Akt), and the tuberous sclerosis protein complex 1/2 (TSC1/2).^28^^,^^29^^,^^30^ Activated mTORC1 phosphorylates p70 ribosomal protein S6 kinase (p70S6K) and eukaryotic initiation factor 4E-binding protein 1 (4EBP1), which promote translation initiation^19^^,^^31^ and subsequently MPS. Insulin signaling reduces MPB via anticatabolic effects.^32^^,^^33^^,^^34^^,^^35^ Insulin modulates MPB through the ubiquitin-proteasomal system and lysosomal autophagy pathway, which act via interactions between Akt/forkhead box O3 (FoxO3) and mTORC1/unc-51 like autophagy activating kinase 1 (ULK1), respectively.^22^^,^^36^^,^^37^

Diverse experimental methods are used to study the physiological and cellular mechanisms of feeding-induced skeletal muscle protein metabolism. Measurements of blood plasma are used to infer the secretion rates of hormones (e.g., insulin) and the rates of absorption of relevant amino acids. The specific amino acids measured vary depending on the ingested solution, but individual amino acids (e.g., leucine, phenylalanine) and general classes of amino acids (e.g., essential amino acids [EAAs], branched chained amino acids) are commonly assessed. Measurements of biopsied muscle are used to assess the post-translational modifications of key proteins in the mTOR signaling cascade (e.g., phospho-Akt, phospho-p70S6K). These phosphorylated proteins are used to infer anabolic signaling; e.g., increased phospho-p70S6K levels imply accelerated MPS. Stable isotope tracers are the primary method used to assess MPS rates. Stable isotopes are added to the nutritional solution consumed by the study participants to determine the rates of incorporation of traced amino acids into skeletal muscle. The enrichment of isotopes in the muscle tissue, measured via serial muscle biopsy samples, is used to estimate the muscle protein synthesized during a specified duration (i.e., the fractional synthetic rate [FSR]). Data regarding stable isotope tracer enrichment in arterial plasma, venous plasma, and muscle are used to estimate the parameters of models of amino acid dynamics (e.g., the 3-pool model^38^^,^^39^). These models enable the inference of transmembrane amino acid transport and muscle protein kinetics across the whole muscle.^38^^,^^39^ While the mechanisms controlling muscle protein metabolism have been identified and characterized, how these mechanisms act as part of an integrated, multi-scale system remain poorly understood. Accordingly, the relative contributions of protein digestion and transport, hormonal signaling, intracellular anabolic signaling, and amino acid metabolism to overall muscle protein metabolism have yet to be quantified.

Mathematical models are powerful tools for analyzing complex biological systems. Models enable the integration of chemical and physical principles, prior knowledge, and experimental data in a coherent framework.^40^ Mathematical models have been developed for aspects of protein metabolism and anabolic signaling. For example, the mechanisms controlling the ultradian secretion of insulin from the pancreas have been studied using models that incorporate the negative feedback loops between insulin and glucose.^41^^,^^42^ Models of amino acid dynamics have expanded upon the previously mentioned 3-pool model and include a six-compartment model of intracellular muscle kinetics of leucine and its transamination product ⍺-ketoisocaproic acid (KIC) across the human forearm^43^ and a ten-compartment model of human protein kinetics incorporating compartments for leucine, KIC, and bicarbonate.^44^ Models of mTORC1 signaling have investigated the control of mTOR by amino acids and insulin,^45^ the control of mTOR complex 2 (mTORC2) in response to amino acid and insulin stimulation,^46^ the influence of AMP-activated protein kinase in response to amino acid and insulin stimulation on downstream mTORC1 activity,^47^ and novel amino acid inputs to the mTOR network.^48^ However, an integrated model of muscle protein metabolism combining insulin secretion, amino acid dynamics, and mTORC1 signaling has yet to be proposed.

The purpose of this study was to develop and analyze a kinetic model of skeletal muscle protein metabolism. The model focused on leucine dynamics because it is the primary amino acid governing MPS.^12^^,^^49^^,^^50^^,^^51^ A compartmental kinetic model is proposed that simulates leucine incorporation into skeletal muscle following ingestion of leucine downstream of core signaling and metabolic processes. The model satisfactorily simulates leucine-mediated signaling and protein metabolism dynamics following bolus and pulsatile feeding. We analyzed the model via simulations to demonstrate that the total levels of p70S6K are a primary determinant of MPS, more so than changes in phospho-p70S6K levels, insulin signaling affects net muscle protein balance through inhibition of MPB, and p70S6K-mediated feedback of mTORC1 signaling reduces MPS in a dose-dependent manner.

## Results

### Model development history

**Figure 1:**
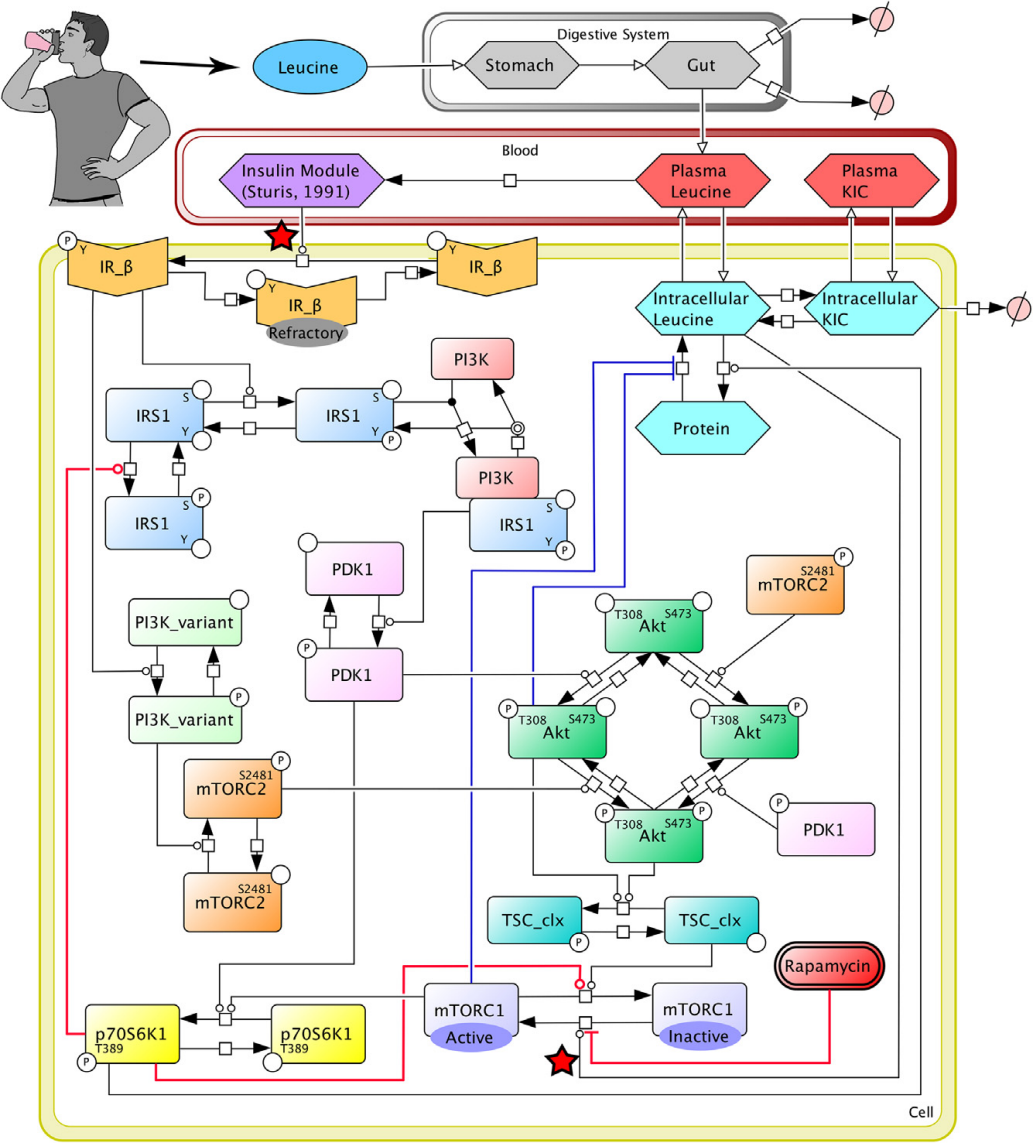
Reaction diagram of the model Reaction diagram for the kinetic model of leucine-mediated signaling and protein metabolism. mTORC2^S2481^ and PDK1^P^ are represented as independent species for the phosphorylation of Akt^S473^ and Akt^S473,T308^, respectively, but both are subject to the same control as the integrated species. The insulin module is presented as a single species in the model diagram, but it is expanded in the model code to include the three species and four negative feedback loops outlined by Sturis et al.^41^ The transfer of masses is denoted by open-headed arrows. Chemical reactions are denoted by solid-headed arrows. Inhibition is denoted by a flat-headed line. Red stars denote the locations of simulated signaling knockdown. Red lines indicate inhibitory links via p70S6K-mediated feedback or rapamycin. Blue lines indicate signaling-mediated control of MPB. P = phospho residue, S = serine, T = threonine, Y = tyrosine.

The development of our kinetic model of leucine-mediated signaling and protein synthesis was an iterative process wherein we added modules, adjusted reactions, meta-analyzed datasets, and added or removed molecules to arrive at a comprehensive and best-fit model. Our model development culminated in the model topology presented in Figure 1.

### Model calibration

**Figure 2:**
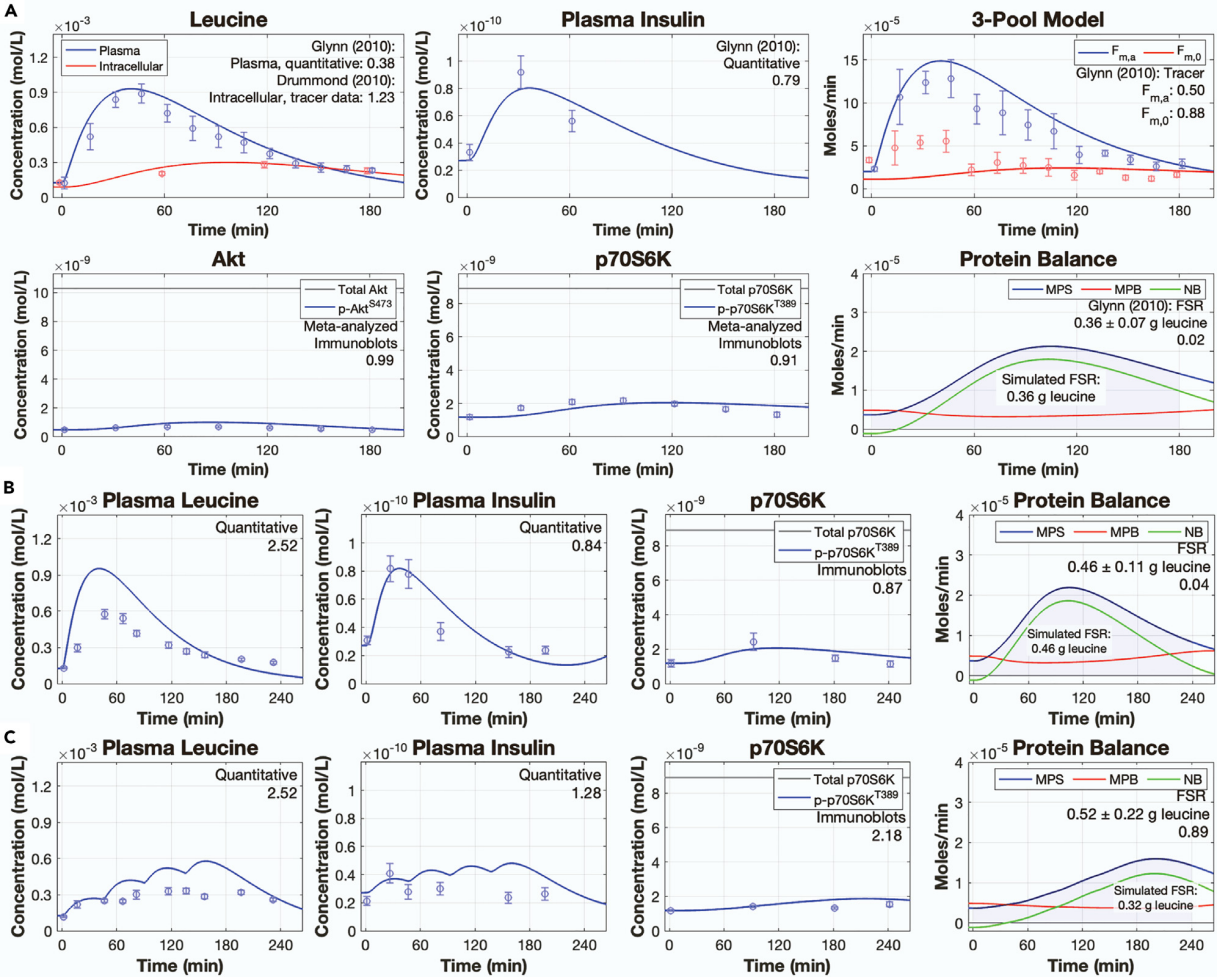
Model calibration and validation (A) Simulated time courses following model calibration of plasma leucine, intracellular leucine, plasma insulin, the three-pool model parameters F_m,a_ and F_m,0_, Akt (total and serine phosphorylated), p70S6K (total and phosphorylated), and muscle protein balance following a 3.5-gram bolus of leucine. Data points represent experimental data collected from two studies following the ingestion of a 3.5-gram bolus of leucine in human subjects.^52^^,^^53^ The data points for phospho-Akt^S473^ and phopsho-p70S6K^T389^ were predicted from spline regression equations obtained from meta-analyzed data. (B and C) Simulated time courses of plasma leucine, plasma insulin, p70S6K, and muscle protein balance following either (B) a single 3.59-gram bolus of leucine^54^ or (C) pulsatile leucine feedings (0.59-grams of leucine provided at 0, 45, 90, and 135 min).^54^ Data points represent experimental data collected from the Mitchell et al.^54^ single bolus intervention (B) or pulsatile feeding intervention (C). Root-mean-square values for each time course are included within each plot. The measured data are presented as means ± SE. FSR = fractional synthetic rate, MPS = muscle protein synthesis, MPB = muscle protein breakdown, NB = net balance. See also Figures S1–S6 and Tables S1–S8.

We calibrated the model parameters in two steps: 1) the parameters from the insulin secretion module were independently fit to the insulin time course (k51-67; volume-specific parameters were not adjusted) and 2) the remaining model parameters were fit to all eight experimental datasets. Calibration of the model parameters resulted in a root-mean-square value of 5.71. The model simulations qualitatively agreed with experimental data for plasma leucine, intracellular leucine, plasma insulin, phospho-Akt^S473^, phospho-p70S6K^T389^, 3-pool parameters (i.e., F_m,a_, F_m,0_), and leucine incorporation to protein in response to a 3.5-gram bolus of leucine (Figure 2A).

### Model validation

We comprehensively validated the model by comparing the output of the calibrated model against data from one pulsatile feeding protocol^54^ and five bolus feeding protocols.^12^^,^^52^^,^^53^^,^^54^^,^^55^ The pulsatile feeding protocol was simulated by providing four 0.9-gram doses of leucine at 45-min intervals, whereas the bolus feeding protocols were simulated by providing a 1.85-gram, 3,42-gram, 3.5-gram, or 3.59-gram bolus of leucine at 0 min. The pulsatile feeding protocol was distinct from the bolus feeding protocol that gave rise to the calibration data, such that it served as a stringent test for model validation. The model predictions for all six validation datasets qualitatively agreed with plasma leucine, intracellular leucine, plasma insulin, phospho-Akt^S473^, phospho-p70S6K^T389^, and MPS (Figures 2B, 2C, and S1).

### The model reveals discrepant experimental measurements

Our model highlighted discrepancies in experimental time course data following leucine ingestion, in particular those pertaining to phospho-p70S6K^T389^ and plasma leucine. As discussed in the STAR Methods, substantial differences existed in the estimates of phospho-p70S6K^T389^ dynamics from different lab groups. Figures S1A and S1F and the experimentally measured data from the Glynn et al. increased-leucine-concentration group^53^ show substantially increased phospho-p70S6K^T389^ at 60 min following leucine ingestion (12.5-, 14.2-, and 39.5-fold increase, respectively) that the model was unable to replicate. Comparatively, Figures S1B‒S1D show lower peak phospho-p70S6K^T389^ levels (2.1-, 1.3-, and 1.6-fold increase, respectively) that the model accurately replicated. We searched for possible methodological differences or covariates to explain the discrepancies between the datasets but were unable to find any.

Discrepancies were also observed in the plasma leucine time courses. We simulated the model against nine experimentally measured time courses, in which the only change introduced to the model was the amount of leucine provided at time 0, which corresponded to the dose provided in the associated experimental dataset. The model accurately simulated the plasma leucine time course in the calibration dataset^53^ and in three validation datasets^52^^,^^53^^,^^55^ (Figures S2A‒S2D). Each of these four interventions involved participants consuming a 10-gram bolus of EAA but with varying leucine content (1.8- to 3.5-grams). In contrast, the model overestimated the plasma leucine levels when leucine was administered in a larger, 15-gram EAA bolus^54^ (Figure S2E) and when leucine was administered in isolation^12^ (Figure S2F). When whey protein^56^^,^^57^ or egg protein^58^ was used as the feeding intervention, the model overestimated the plasma leucine levels and predicted an earlier peak (Figures S2G‒S2I), thus suggesting that the digestion and absorption kinetics are modulated when whey or egg protein is ingested compared to EAA alone. The amino acid compositions for each of the EAA interventions are listed in Table S8.

### Model analysis: simulation of unobserved variables

Using the validated model, we analyzed the dynamics of model variables that are typically unmeasured in experimental studies (Figure 2, protein balance panels; Figure S3). For example, mTORC1 activity is difficult to measure experimentally. The mTORC1 kinase is regulated by the small GTPase, Ras homolog enriched in brain (Rheb).^59^ Following leucine feeding, Rheb dissociates from TSC (TSC-Rheb, inhibitory state) allowing for mTORC1-Rheb colocalization, thereby increasing mTORC1 activity.^59^ Quantification of the mTORC1-Rheb colocalization requires assays that measure protein proximity, such as immunofluorescence,^60^ and these methods have seldom been employed to date in skeletal muscle metabolism research. In addition, the phosphorylation of the Ser2448 residue has been commonly but incorrectly measured as a proxy for mTORC1 kinase activity.^61^ The model can simulate mTORC1 activity following leucine feeding and shows that little change in mTORC1 activity is required to induce the observed changes in MPS (Figure S3).

MPS and overall muscle protein dynamics are commonly inferred by the FSR in wet-lab experiments. However, FSR only accounts for MPS but not MPB, such that using FSR alone to infer the total muscle protein balance is predicated on the assumption that little to no change in MPB occurs following feeding. The model simulates MPS, MPB, and net balance (NB) following leucine intake, thus obviating the need to assume that MPS accounts for all changes in muscle protein balance. The model confirms that the MPS response following leucine feeding exceeds that of MPB but that MPB is dynamic following feeding. Specifically, MPB decreases immediately after feeding in response to increased phospho-Akt^T308^ and active mTORC1 concentrations but then increases in the hours following feeding when phospho-Akt^T308^ and active mTORC1 concentrations return to post-absorptive levels (Figure 2: protein balance panel). Prior to feeding, NB was negative, indicating net MPB. Post feeding, the combination of increased MPS and decreased MPB causes NB to be positive for several hours following feeding, corresponding to net MPS (Figure 2, protein balance panels).

### Model analysis: knockdown of leucine signaling impairs muscle protein metabolism

**Figure 3:**
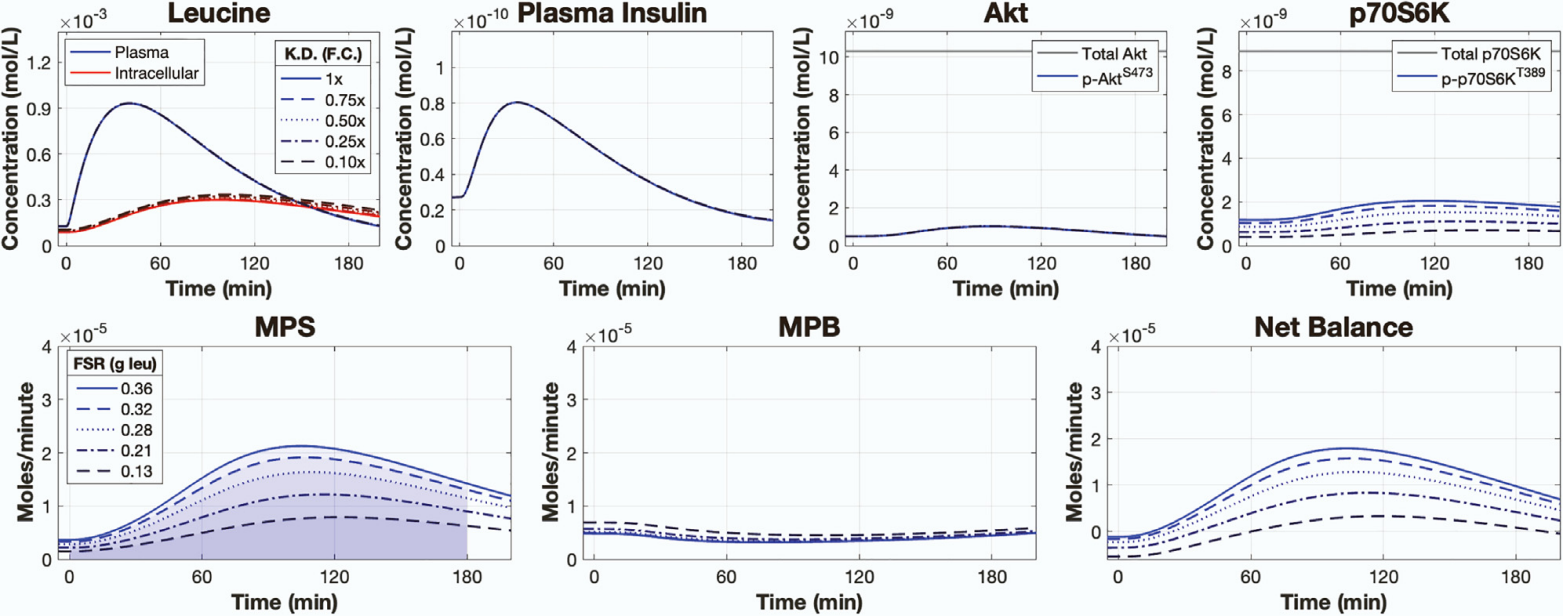
Knockdown of leucine signaling impairs MPS and net balance Simulated time courses of plasma leucine, intracellular leucine, plasma insulin, Akt, p70S6K, and muscle protein balance following a 3.5-gram bolus of leucine with varying degrees of knockdown to the rate controlling leucine-mediated mTORC1 activity. Signaling knockdown was simulated by decreasing the kinetic rate parameter controlling leucine-mediated mTORC1 activity by 1×, 0.75×, 0.50×, 0.25×, and 0.10× of its calibrated value. F.C. = fold change, FSR = fractional synthetic rate, K.D. = knockdown, MPS = muscle protein synthesis, MPB = muscle protein breakdown. See also Figures S4 and S5.

Knockdown of leucine-mediated mTORC1 signaling caused a substantial loss of MPS (Figure 3). We simulated the knockdown of leucine-mediated mTORC1 activity by reducing the rate parameter controlling leucine-mediated mTORC1 activation (k39) by 1×, 0.75×, 0.50×, 0.25×, and 0.10× its calibrated value. All other model parameters remained the same. These adjusted models were then simulated using a single 3.5-gram bolus of leucine as input. Knockdown of mTORC1 activity reduced the downstream phosphorylation of p70S6K^T389^, which resulted in a proportional loss of MPS. The knockdown of mTORC1 activity had little effect on MPB, such that there was a reduced NB that corresponded to the loss in MPS.

Knocking down leucine-mediated mTORC1 activity mimics the effects of rapamycin, a specific mTORC1 inhibitor in animals^13^ and humans,^55^ which inhibits the early amino acid- and resistance training-induced increases in protein synthesis in humans.^62^ To help validate our simulations and show the model’s potential usefulness for exploring rapamycin pharmacodynamics, we tested the model against the data from Dickinson et al.^55^ We simulated the model following the ingestion of a 1.8-gram bolus of leucine while progressively knocking down the rate controlling leucine-mediated mTORC1 activity (k39) until we obtained a qualitative match to the Dickinson et al.^55^ dataset. We found that knocking down the rate controlling leucine-mediated mTORC1 activity to 0.05× of its calibrated value accurately simulated the loss of MPS and p70S6K signaling following leucine ingestion with rapamycin treatment (Figure S4). The loss of MPS was mediated by the loss of both post-absorptive and leucine-mediated phospho-p70S6K^T389^ activity.

### Model analysis: post-absorptive and fed-state leucine-mediated signaling both contribute to controlling muscle protein metabolism

**Figure 4:**
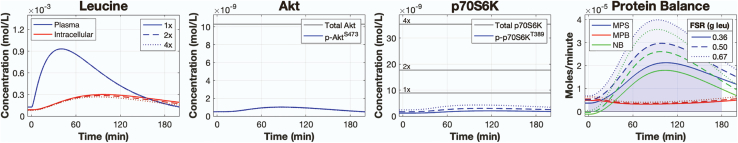
Increasing post-absorptive p70S6K levels substantially increases MPS Simulated time courses of plasma leucine, intracellular leucine, Akt, p70S6K, and muscle protein balance following a 3.5-gram bolus of leucine with the non-phosphorylated p70S6K concentration at the calibrated value (1×), two times the calibrated value (2×), and four times the calibrated value (4×). FSR = fractional synthetic rate, MPS = muscle protein synthesis, MPB = muscle protein breakdown, NB = net balance.

In analyzing the model, we noticed that changes to total p70S6K levels influenced MPS. We pursued this observation by increasing the concentration of non-phosphorylated p70S6K by factors of two (2×) and four (4×) and simulating the model in response to a 3.5-gram bolus of leucine with all other parameters unchanged. When the concentration of non-phosphorylated p70S6K was increased by a factor of two or four, MPS respectively increased 139% or 185% (Figure 4). The increase in p70S6K concentration produced a marked increase in total phospho-p70S6K^T389^ signaling as measured by the area under the curve (AUC; 1×: 3.15 × 10^−7^ mol·min/L, 2×: 4.63 × 10^−7^ mol·min/L, 4×: 6.65 × 10^−7^ mol·min/L). The change in p70S6K concentration did not affect MPB, such that there was an increased NB in both simulations that corresponded to the increase in MPS.

Following the finding that total p70S6K levels exert a strong influence on the MPS response, we tested whether leucine-mediated signal activation was necessary to produce a full MPS response when phospho-p70S6K levels were sustained at post-absorptive levels. The model was simulated with p70S6K levels maintained at their post-burn-in values and with mTORC1-mediated p70S6K phosphorylation inhibited following feeding. A 33% reduction in the MPS response was observed when mTORC1-mediated p70S6K phosphorylation was inhibited, with 0.24 g of leucine incorporated in the phospho-p70S6K-inhibited condition, compared to 0.36 g of leucine incorporated in the phospho-p70S6K responsive condition (Figure S5).

**Figure 5:**
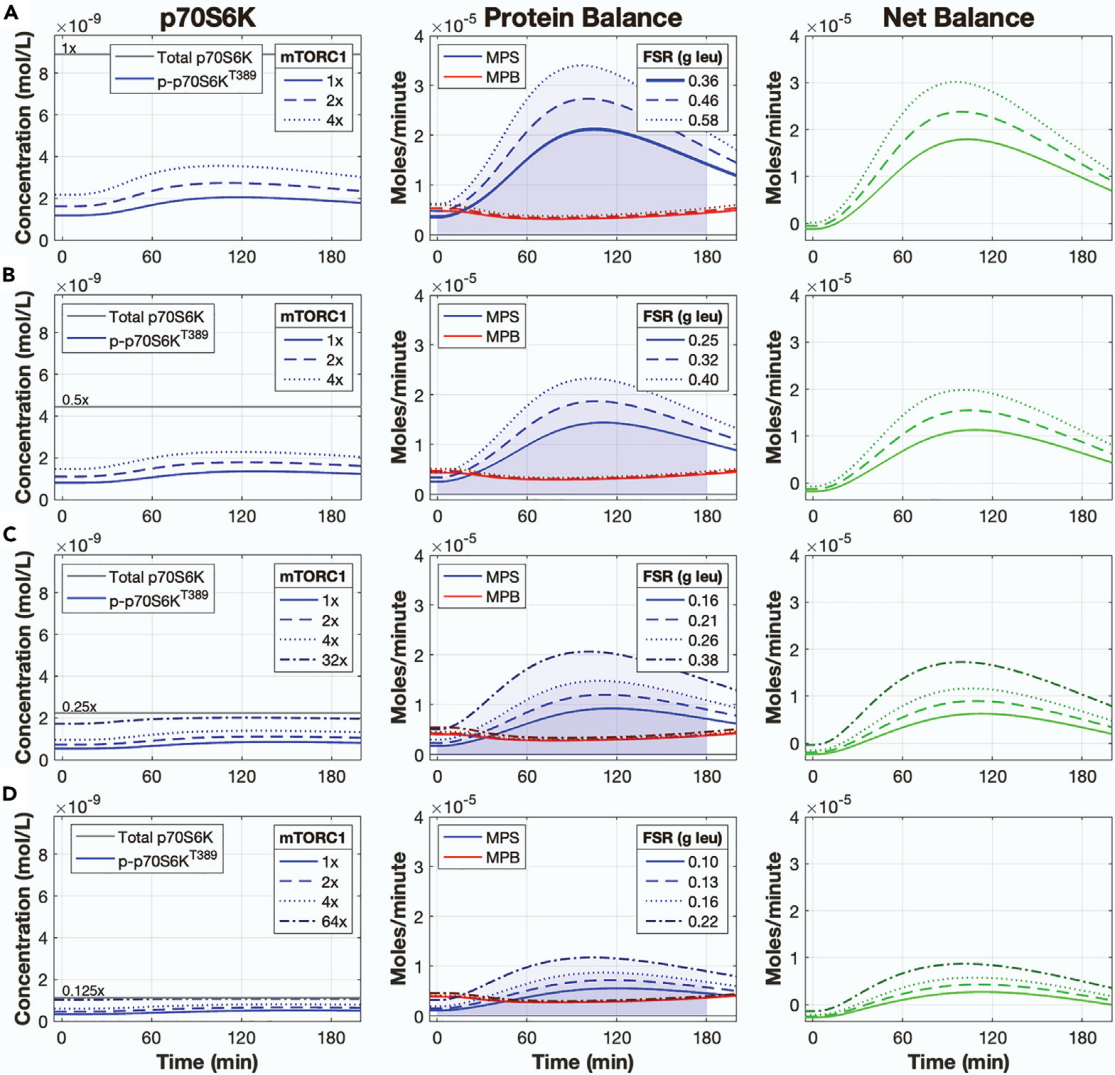
Enhanced signal activation can compensate for moderate losses in p70S6K levels Simulated time courses of p70S6K, MPS, MPB, and NB following a 3.5-gram bolus of leucine with total p70S6K concentrations (p70S6K + phospho-p70S6K^T389^) at (A) the calibrated value (1×), (B) 0.5-times the calibrated value (0.5×), (C) 0.25-times the calibrated value (0.25×), and (D) 0.125-times the calibrated value (0.125×). At each level of p70S6K, the rate controlling the mTORC1 kinase was simulated at 1×, 2×, and 4× of its calibrated value. The 0.25× and 0.125× p70S6K concentration were additionally simulated with the rate controlling mTORC1 kinase set at 32× and 64×, respectively. The baseline MPS time course (1× p70S6K, 1× mTORC1 kinase) in (A) was bolded to serve as a reference line for (B), (C), and (D). FSR = fractional synthetic rate, MPS = muscle protein synthesis, MPB = muscle protein breakdown, NB = net balance.

These results suggested that MPS is determined by both total p70S6K levels and additional phosphorylation of p70S6K mediated by mTORC1 following feeding. The potential thus exists for one to compensate for the other. Indeed, reducing total p70S6K levels (i.e., both p70S6K and phospho-p70S6K) by factors of 0.5 (0.5×), 0.25 (0.25×), or 0.125 (0.125×) caused respective reductions in MPS by 31%, 56%, and 72% (1×: 0.36 g leucine, 0.5×: 0.25 g leucine, 0.25×: 0.16 g leucine, 0.125×: 0.10 g leucine; Figure 5). However, independently increasing the rate controlling mTORC1-mediated p70S6K phosphorylation (k40) by 4× and 32× in the 0.5×- and 0.25×-p70S6K-level simulations, respectively, restored the MPS response (1× p70S6K, 1× mTORC1 kinase rate: 0.36 g leucine; 0.5× p70S6K, 4× mTORC1 kinase rate: 0.40 g leucine; 0.25× p70S6K, 32× mTORC1 kinase rate: 0.38 g leucine; Figures 5B and 5C). However, the MPS response was not restored in the 0.125×-p70S6K-level simulation even when mTORC1 kinase activity was increased by 64×, which produced a nearly saturated phospho-p70S6K^T389^ response (1× p70S6K, 1× mTORC1 kinase rate: 0.36 g leucine; 0.125× p70S6K, 64× mTORC1 kinase rate: 0.22 g leucine; Figure 5D). As with the previous simulations, no changes in MPB occurred with changes to the p70S6K levels or mTORC1 kinase activity, such that the changes in NB corresponded exclusively to the changes in MPS.

### Model analysis: the roles of insulin signaling in controlling muscle protein metabolism

**Figure 6:**
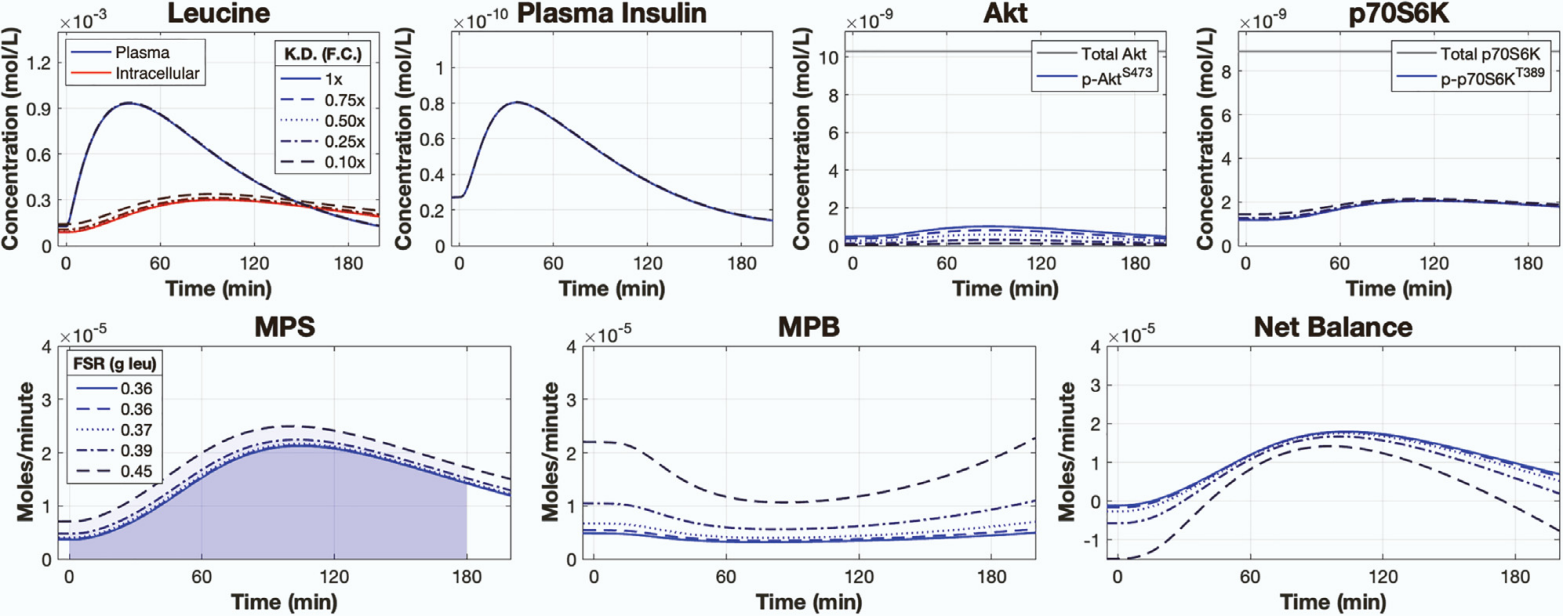
Knockdown of insulin signaling reduces net protein balance Simulated time courses of plasma leucine, intracellular leucine, plasma insulin, Akt, p70S6K, and muscle protein balance following a 3.5-gram bolus of leucine with varying amounts of knockdown to the rate controlling insulin-mediated insulin receptor (IR_β_) phosphorylation. Signaling knockdown was simulated by modulating the kinetic rate parameter by 1×, 0.75×, 0.50×, 0.25×, and 0.10× its calibrated value. F.C. = fold change, FSR = fractional synthetic rate, K.D. = knockdown. MPS = muscle protein synthesis, MPB = muscle protein breakdown.

Leucine and insulin both positively regulate mTORC1 activity and downstream MPS;^18^ however, the contribution of insulin signaling to leucine-mediated MPS is unclear. Some studies suggest that insulin is required to induce a maximal MPS response,^63^^,^^64^ whereas others propose that it is not.^34^^,^^65^ To evaluate the contribution of insulin signaling on leucine-mediated MPS, we knocked down insulin signaling in the model in varying amounts (1×, 0.75×, 0.50×, 0.25×, 0.10×) and compared the resulting changes in muscle protein balance following the ingestion of a 3.5-gram leucine bolus. The knockdown was achieved by reducing the rate controlling insulin-mediated IR_β_ phosphorylation (k16) while keeping all other model parameters unchanged. We observed reduced phospho-Akt but no corresponding decreases in phospho-p70S6K or MPS (Figure 6). In fact, the model predicted a slight increase in phospho-p70S6K and MPS when insulin signaling was knocked down. However, the loss of insulin signaling resulted in a substantial increase in MPB since there was a loss of phospho-Akt^T308^-mediated inhibition of MPB. The increase in MPB contributed to a moderate increase in intracellular leucine content, which caused the elevated phospho-p70S6K and MPS. The increased MPB surpassed the relatively small increase in MPS, leading to reduced NB as measured by the AUC (NB AUC, 1×: 0.27 g leucine; 0.75×: 0.26 g leucine; 0.5×: 0.25 g leucine; 0.25×: 0.22 g leucine; 0.1×: 0.11 g leucine).

### Model analysis: the contributions of the p70S6K feedback pathways on muscle protein balance

**Figure 7:**
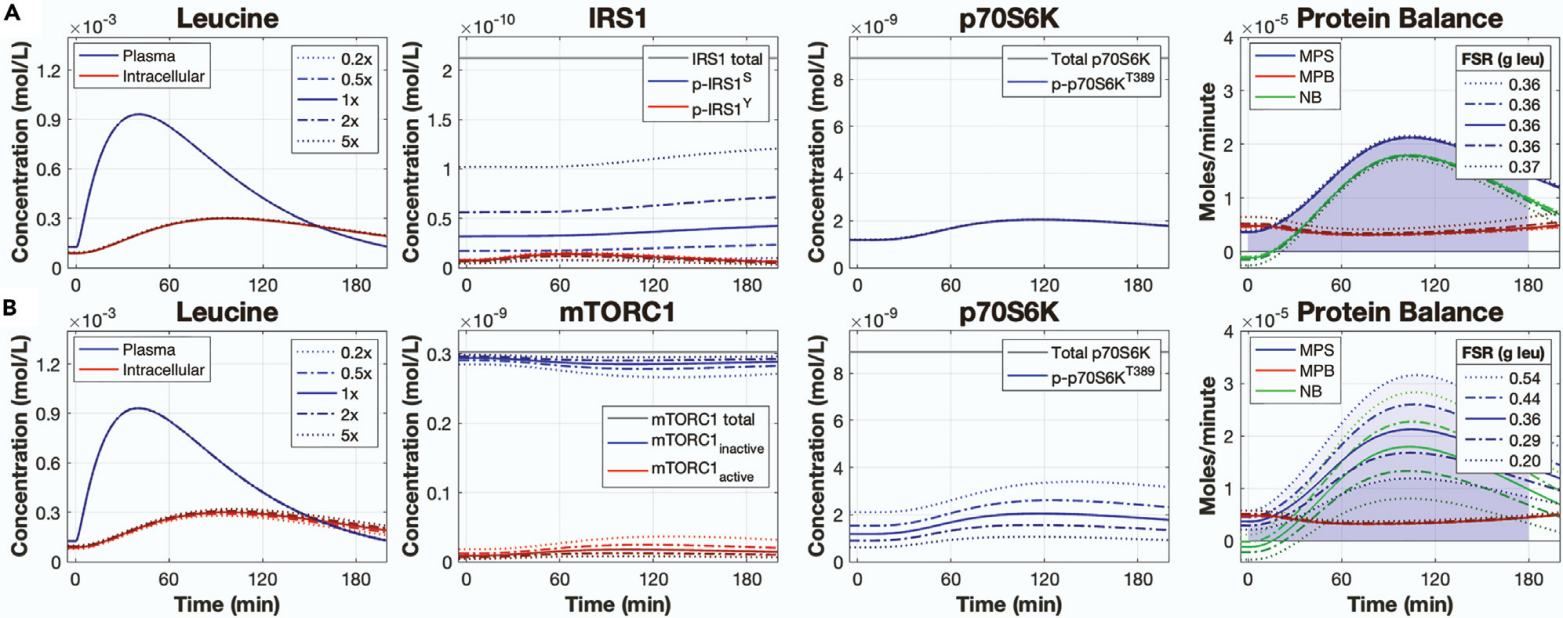
Contributions of phospho-p70S6K^T389^-mediated negative feedback on muscle protein balance Simulated time courses following a 3.5-gram bolus of leucine with the kinetic parameters controlling (A) phospho-p70S6K^T389^-mediated phosphorylation of the IRS1 serine residue (k21) and (B) phospho-p70S6K^T389^-mediated inhibition of mTORC1 activity (k43) simulated at 0.2×, 0.5×, 1×, 2×, and 5× their calibrated value. Total IRS1 includes the PI3K phospho-IRS1^Y^ complex and the non-phosphorylated IRS1 protein, neither of which are presented in the IRS1 plot. FSR = fractional synthetic rate, MPS = muscle protein synthesis, MPB = muscle protein breakdown, NB = net balance.

Phospho-p70S6K^T389^ participates in two negative feedback pathways: one that promotes the phosphorylation of the serine residue on IRS1 and another that acts on mTORC1 to impair its kinase activity. We investigated the contributions of these feedback pathways to muscle protein balance. Specifically, we increased and decreased the kinetic parameters controlling phospho-p70S6K^T389^-mediated phosphorylation of the IRS1 serine residue (k21) and phospho-p70S6K^T389^-mediated inactivation of mTORC1 (k43) by 0.2×, 0.5×, 1×, 2×, and 5× and assessed the changes in muscle protein balance following the ingestion of a 3.5-gram leucine bolus. We found that increasing or decreasing k21 produced reciprocal changes in phospho-IRS1^S^, but muscle protein balance remained unchanged (Figure 7A). In contrast, adjusting k43 caused changes to mTORC1 activity and MPS. Specifically, increasing the strength of negative feedback on mTORC1 reduced MPS while decreasing it led to increased MPS (Figure 7B).

## Discussion

Skeletal muscle metabolism is complex and involves the dynamic interplay of multiple physiological processes operating at different levels of organization. Experimental studies have examined these processes in relative isolation from one another, such that how these processes operate as a system remains incompletely understood. In this study, we developed and analyzed a kinetic model of protein translational signaling and protein metabolism in human skeletal muscle cells in response to leucine ingestion. Our primary objective was to create a first-generation model of the signaling controlling protein metabolism in human skeletal muscle that incorporated physiologically realistic input dynamics to drive the downstream protein signaling and metabolic responses. We accomplished this goal by modifying and amalgamating published models of mTOR signaling,^46^ skeletal-muscle leucine kinetics,^43^ and insulin dynamics^41^ and then updating the overall model topology according to the latest literature. The resulting model satisfactorily predicts data collected from human participants in response to various leucine feeding interventions. Our model revealed three key findings: 1) total levels of p70S6K are an important determinant of MPS rates, the contribution of which was greater than the additional phospho-p70S6K in response to feeding; 2) insulin signaling influences muscle protein balance through its inhibition of MPB; and 3) p70S6K-mediated negative feedback of mTORC1 signaling reduces MPS in a dose-dependent manner. Our study thus motivates new hypotheses regarding the mechanism by which signaling influences protein metabolism, reconciles controversial aspects of protein metabolism, and provides a foundation for future modeling studies of skeletal muscle protein metabolism.

### Main findings and their implications

A key finding from our model analysis was that the absolute levels of p70S6K substantially influenced MPS. Most experimental studies assess the relative differences in phospho-p70S6K levels between interventions and use these data to infer MPS. Although phospho-p70S6K controls MPS^31^^,^^55^ and our model confirms the need for phospho-p70S6K signaling for MPS (Figures 5 and S5), our results suggest that differences in total levels of p70S6K may have a more pronounced influence on MPS than the enhancement caused by leucine-stimulated mTORC1 signaling following feeding. Of the experimental studies we found that featured amino acid or whey protein feedings, none quantified the absolute concentrations of signaling proteins (phosphorylated or non-phosphorylated).

This result has important health implications because differences in total p70S6K levels may determine an individual’s susceptibility to sarcopenia and responsiveness to resistance training. Sarcopenia is thought to be primarily due to *anabolic resistance* (reduced MPS response) to feeding.^22^ Our results suggest that a cause of anabolic resistance may be reduced p70S6K protein levels. Indeed, Cuthbertson et al.^66^ found that the total concentration of p70S6K protein in elderly men was 50% of that in young men, and this corresponded to ∼30%–40% less myofibrillar FSR in the elderly men following EAA feeding. In addition, our model suggests that modest losses in p70S6K protein levels can be compensated for by increased mTORC1 kinase activity. This scenario appears to be supported from two experimental studies. Markofski et al.^67^ found an increase in post-absorptive p70S6K^T389^ phosphorylation in the older adults group but no difference in post-absorptive FSR between young and older adults. Cuthbertson et al.^66^ observed a statistically non-significant increase in post-absorptive p70S6K^T389^ phosphorylation in the elderly group and in response to low-dose EAA feedings (10 g). The increased post-absorptive p70S6K^T389^ phosphorylation in the elderly group potentially allowed for the similar MPS responses between the old and young groups. By comparison, resistance training induces an anabolic state wherein MPS is elevated beyond MPB to allow for an increase in NB and muscle mass.^22^ Studies in rats show that the levels of several mTOR signaling proteins, including p70S6K, may be increased following chronic resistance training when biopsies are extracted following the recovery from exercise (i.e., 24–28 h post exercise bout).^68^^,^^69^ We postulate that the increase in these mTOR signaling proteins may contribute to the increase in MPS and the resulting increase in muscle mass.

Overall, these results suggest that an individual’s anabolic signaling system can mechanistically act in one of two ways to support MPS: 1) a “permissive” mechanism or 2) an “activation-dependent” mechanism. In the “permissive” mechanism, relatively high post-absorptive levels of phospho-p70S6K are sufficient to support MPS when amino acid availability increases. Leucine-mediated activation of mTORC1 contributes to MPS but to a relatively minor extent. In this case, the system is always “primed” for when amino acids are present. In the “activation-dependent” mechanism, post-absorptive phospho-p70S6K levels are relatively low and insufficient to support adequate MPS without additional leucine-stimulated mTORC1 activation.

An important benefit of our model is that it enables the simulation of all species within the system, which enables investigation of species that are commonly overlooked in experimental interventions. For example, MPS, MPB, and muscle protein balance (i.e., NB) are all simulated. The literature often states that MPS is more sensitive to anabolic stimuli compared to MPB and is the primary determinant of changes to muscle protein balance.^21^^,^^70^ Additionally, MPB is more methodologically challenging to measure compared to MPS.^71^ For these reasons, experimental studies commonly feature MPS (i.e., FSR) only, which is used to infer changes to muscle protein balance. However, ignoring MPB will cause one to overlook its contributions to muscle protein balance and may cause misinterpretations regarding the role of insulin. Insulin signaling has two primary roles in the system: 1) it stimulates mTORC1 activity and 2) it inhibits MPB. Controversy exists regarding the influence of insulin on muscle anabolism. Studies in rats document reduced MPS when insulin secretion was blocked^64^ or maintained at post-absorptive levels^63^ following amino acid feeding. In contrast, studies in humans report unchanged MPS in response to casein protein ingestion^65^ or intravenously administered amino acids^34^ alongside insulin clamped at higher-than-systemic post-absorptive concentrations. We sought to assess the role of insulin on muscle anabolism by knocking down insulin signaling in the model. We found that knocking down insulin signaling had little effect on MPS but resulted in increased MPB, which in turn reduced muscle protein balance (Figure 6; NB AUC 1×: 0.27 g leucine; 0.10×: 0.11 g leucine). Therefore, studies in which only MPS was measured may conclude that insulin has little influence on muscle anabolism, whereas considering both MPS and MPB leads to the hypothesis that the increase in MPB following insulin knockdown reduces muscle protein balance to a quantitatively important extent.

We also assessed two p70S6K-mediated negative feedback pathways to better understand their control over muscle protein metabolism. One pathway features p70S6K-mediated phosphorylation of the serine residues of IRS1.^28^^,^^29^^,^^31^ Serine-phosphorylated IRS1 promotes the degradation of IRS1, thereby negatively regulating insulin signaling.^31^ Our analysis of this pathway confirmed that p70S6K controlled the level of serine-phosphorylated IRS1 and exerts slight control of downstream signaling (i.e., phospho-Akt^S473^; data not shown), but this effect did not propagate to meaningful changes in MPS. This result is consistent with our previous result that knockdown of insulin signaling had little effect on MPS, and with other studies that showed that insulin signaling contributes little to MPS.^34^^,^^65^^,^^66^ The other feedback pathway was p70S6K-mediated phosphorylation of the Ser2448 residue on mTOR.^72^ This residue resides in the mTOR negative regulatory domain.^61^ Phosphorylation of the Ser2448 residue reduces mTORC1 kinase activity,^61^^,^^72^^,^^73^^,^^74^ thereby reducing MPS. Our analysis of this feedback loop showed a dose-response relationship with MPS. A poorly understood phenomenon in muscle protein metabolism is the “muscle-full effect,” in which MPS decreases after feeding despite continually elevated plasma and intracellular EAA and leucine levels.^56^^,^^75^ We wonder whether the p70S6K/mTORC1 negative feedback loop could contribute to the muscle-full effect.^56^ This hypothesis could be experimentally tested by inhibiting the p70S6K/mTORC1 negative feedback pathway by blocking the Ser2448 binding site or removing the residues in the mTOR negative regulatory domain, and measuring MPS in response to EAA or whey protein feeding.

### Validity of the results

The robustness of the aforementioned findings is ultimately predicated on the validity of the model, which we took great lengths to establish. The model calibration process integrated several data types, including quantitative data (e.g., plasma leucine, plasma insulin), isotopic tracer data (e.g., intracellular leucine, 3-pool parameters, FSR), and semi-quantitative immunoblots (e.g., phospho-Akt^S473^, phospho-p70S6K^T389^). Due to the semi-quantitative nature of immunoblot data and the variability in immunoblot measurements,^76^ we employed a meta-analytic approach to attempt to increase the accuracies of the phospho-Akt^S473^ and phospho-p70S6K^T389^ time courses (described in the STAR Methods). We used the regression model from the meta-analysis to impute time course data for each species in 30-min increments (i.e., six data points over the 180-min calibration period), which provided time courses of higher resolution for data fitting compared to single experimental time courses. Additionally, we used selected post-absorptive 3-pool model parameter values^39^ and the post-absorptive KIC oxidation rate^44^ to calibrate the post-absorptive skeletal-muscle leucine module.

Once the model was satisfactorily calibrated, we searched extensively for appropriate validation datasets. We located six such datasets, five of which featured single, bolus feedings of varying leucine doses (1.85- to 3.59-grams) and one that featured a pulsatile feeding protocol, i.e., repeated small doses of leucine. The pulsatile feeding intervention served as a particularly stringent test for model validation. By adjusting only the timing and dose of leucine ingestion in the model (i.e., all other model parameters were unchanged) across these heterogeneous interventions (i.e., each study featured variable participant characteristics, differences in ingested solutions, etc.), the model showed a remarkable ability to successfully predict qualitative features such as the timing of peak concentrations or rates and the overall dynamics of the variables measured in the six validation datasets. Several cases existed in which the model simulations differed from the measured data, but these discrepancies were typically slight over- or underestimations and the model predictions still captured the trends in the data. The model showed a strong ability to fit phospho-data time courses, except for two, but even in these cases the model achieved satisfactory error cost values (discussed in the following in the limitations of the study subsection). Furthermore, the model achieved good fits for all intracellular leucine simulations and F_m,a_ simulations.

The model predicted four aspects of the validation data less well, but plausible explanations exist for most of these cases. First, the model overpredicted the plasma leucine concentrations relative to the measured values in three of the six datasets (Figures S1B‒S1D). We propose that these discrepancies were in part due to the different nutrient formulations fed to the participants, the effects of which we discuss in detail in the limitations of the study subsection. Second, in two of the bolus feeding validation datasets,^53^^,^^55^ the phospho-p70S6K^T389^ experimental measurements appeared to be overestimated and exceeded what the model could simulate. We propose that these discrepancies likely arose from methodological differences in data collection and analysis, discussed in detail in the STAR Methods (Meta-analysis of phospho-p70S6K^T389^ and phospho-Akt^S473^ data). Third, in the Glynn et al.^53^ 1.85-gram leucine intervention, a high plasma insulin response at 30-min was observed that the model did not replicate. Leucine is an insulin secretagogue, such that a leucine dose-insulin response relationship should exist.^23^^,^^24^ However, the reported plasma insulin response was excessive compared to other interventions that provided greater leucine doses (3.42-grams,^12^ 3.5-grams,^53^ 3.59 g^54^) but that reported lower plasma insulin levels at 30 min. Fourth, the model underestimated the total leucine synthesized over the intervention period (i.e., an extrapolation of FSR) in three of the validation datasets.^12^^,^^54^^,^^55^ Indeed, substantial variability in experimentally measured leucine synthesis (i.e., FSR) was observed: Glynn et al.^53^ and Dickinson et al.^55^ applied similar feeding interventions (1.85 g leucine, 10 g EAA and 1.8 g leucine, 10 g EAA, respectively) but reported markedly different amounts of leucine synthesized, i.e., 0.11 ± 0.02 g at 180 min versus 0.32 ± 0.07 g at 120 min, respectively. The underestimation of FSR could in part be because of leucine being the only amino acid considered in the model. Although leucine is the most potent amino acid in activating mTORC1 and downstream MPS,^12^^,^^13^^,^^14^^,^^15^^,^^77^ many of the simulated interventions provided a mixed EAA solution to participants, and the other EAAs (e.g., Phe, Thr) can contribute to the activation of mTORC1 and MPS.^78^ We decided to focus on leucine exclusively to balance model accuracy with parsimony, justified by leucine’s dominant potency compared to the other amino acids. So, while the model features instances of lack of fit, on balance it accurately replicates the biological responses to various leucine feeding interventions.

### Practical implications

Our study represents the first model that simulates skeletal muscle protein metabolism in humans using realistic whole-body dynamics of leucine and insulin following feeding. Previous mathematical models of translational signaling and protein metabolism are limited to cultured cell lines (e.g., HeLa cells, C2C12 myoblasts, CHO cells) and used non-physiological input dynamics (e.g., constant inputs). Therefore, our model has the advantage of being directly applicable to human skeletal muscle. Furthermore, our model can simultaneously fit, and therefore reconcile, data from distinct experimental methods, e.g., liquid chromatography-based measures of plasma amino acid levels, ELISA-based measures of plasma hormone levels, immunoblotting of signaling proteins, and metabolite fluxes from stable isotope tracers. The integration of various data types into a single framework enables the clear representation of the multi-scale system through which leucine functions and can act as a tool for understanding the complex components of skeletal muscle metabolism (e.g., 3-pool model, leucine kinetics). Findings from our model analysis suggest the need to quantify the absolute total and phospho-protein levels of key signaling proteins (e.g., p70S6K) to better understand their role in MPS control. These differences in protein levels may contribute to differences in MPS rate, which could lead to the development of sarcopenia. Additionally, our model highlights the lack of phospho-protein time course data for many proteins of the mTOR signaling network following feeding interventions. Although phospho-Akt and phospho-p70S6K can inform much of the insulin- and leucine-induced signaling activity, the measurement of additional phospho-proteins throughout the signaling network following human interventions would allow for a better understanding of the overall network dynamics.

### Conclusions

In summary, we have developed and analyzed a mathematical model of protein translational signaling in human skeletal muscle cells following leucine feeding that features hormonal and nutritional inputs. Our findings suggest that total levels of p70S6K have a key influence on MPS rates that is greater than that of dynamic changes in phospho-p70S6K levels, that insulin signaling plays a prominent role in muscle protein balance through its effects on MPB and NB, and that p70S6K-mediated feedback on mTORC1 may restrict MPS. Our model provides an essential tool for integrating diverse data types, reconciling contradictory data, and systematically investigating the various multi-level mechanisms governing skeletal muscle protein metabolism. The model may therefore be used to generate more informed and sophisticated hypotheses for experimental testing.

### Limitations of the study

Any model must balance comprehensiveness with parsimony, and we discuss here four of our major modeling decisions. First, we made the simplifying assumption to focus the model exclusively on leucine, despite leucine being just one of 20 amino acids that comprise human proteins. Leucine was the focus because it is the most important amino acid with respect to anabolic signaling,^12^^,^^13^^,^^14^^,^^15^ and including other amino acids would have greatly increased the model’s complexity. The impacts of this decision cannot be definitively evaluated at present, but our results indicate that discrepancies may be introduced at the level of leucine digestion and absorption. Specifically, we compared our model to data from feeding interventions featuring differing nutrient compositions, including leucine alone, EAA solutions, and whey or egg protein. In the case of whey and egg protein, the model overestimated the plasma leucine response with earlier peak concentrations in each dataset (Figures S2G‒S2I). This finding makes sense because both protein sources consist of whole proteins (whey is a component of whole milk), which require digestion unlike the free-form amino acids in EAA. Thus, leucine absorption is delayed when administered in whey protein.^12^^,^^79^ Simulation of more complex protein sources would require more details regarding protein digestion and absorption,^80^^,^^81^^,^^82^ which represents a future direction for our model. In addition, the model was able to accurately simulate four plasma leucine time courses following varying leucine boluses (1.80–3.50 g) provided as part of a 10-gram EAA solution (Figures S2A‒S2D). However, the model overestimated the plasma leucine response in two datasets, one that provided 3.59-grams of leucine with 15-grams of EAA^54^ (Figure S2E) and another that provided 3.42-grams of leucine alone^12^ (Figure S2F). In accordance with the whey protein results, the greater amount of EAA in Mitchell et al.^54^ may have reduced the amount of leucine absorbed, thus causing the overprediction (Figure S2E). However, our model should have underpredicted the plasma leucine data of Wilkinson et al.^12^ but instead it overpredicted them (Figure S2F). Collectively, these results indicate that different nutrient formulations may affect leucine absorption dynamics, but lab-specific differences in experimental methods and random variability may also affect plasma leucine data. Future versions of the model will examine the effects of the other feeding interventions.

Second, the model does not simulate the muscle-full effect. Maximal MPS rates occur following the ingestion of ∼10-grams of EAA^57^^,^^66^^,^^83^^,^^84^ every 3 h^83^; increasing the feeding dose or frequency does not further increase MPS. Therefore, the current model may overestimate MPS following large or frequent doses of leucine. Although this issue may be problematic when attempting to use the model to determine optimal feeding profiles, we do not believe that any interventions simulated within this study contained sufficiently high doses of leucine to elicit the muscle-full effect, such that this limitation should not influence our findings.

Third, we made the simplifying assumption to use ordinary differential equations (ODEs) to encode the model, which requires the assumption that all biochemical reactions occur deterministically in a homogeneous compartment and do not account for spatially distributed cellular processes. Regarding protein localization, both the insulin- and leucine-dependent pathways interact with proteins bound to the surface of lysosomes (e.g., Rheb, Ragulator) prior to activating mTORC1.^26^^,^^85^ To account for spatially localized species, we have assumed the model to be a well-mixed compartment and we have modeled any changes in species localization (i.e., movement between the blood plasma and cellular space) with ODEs through elementary reactions between different compartments, resolving the requirement to model changes in concentration with respect to space.^40^

The fourth decision we made was to retain most of the components of the mTOR signaling network, which made the model relatively complex. Complex models can be limited in several ways, such as challenges in estimating the parameter values, overfitting the data, and reduced tractability. Some may argue that a more parsimonious model would be better. We justify the model’s scope as follows. First, we emphasize that we did simplify parts of the model, in particular removing proteins that did not influence model dynamics (e.g., PRAS40). Second, many of the represented reactions depict “lumped” processes in which distinct molecular processes are collectively represented by one rate equation. For example, the mechanisms of leucine sensing are implicitly represented within reaction 39 (Table S2) and the mechanisms regulating translation initiation via the mTORC1-mediated phosphorylation of p70S6K and 4EBP1 *in vivo* have been simplified to the mTORC1-mediated phosphorylation of p70S6K and the p70S6K-mediated regulation of MPS. Therefore, the model is relatively simple compared to biological reality. Third, the experimental data used for model calibration featured model components that were distributed throughout the model topology, including at the input (e.g., plasma leucine, insulin), leucine kinetics (e.g., intracellular leucine, 3-pool model), central signaling network (phospho-Akt^S473^), downstream signaling level (phospho-p70S6K^T389^), and final output (MPS). Therefore, we could determine whether the model was operating correctly and, if not, at what level the model failed to replicate the experimental data.^86^ Furthermore, there was no evidence of model overfitting; rather the model still exhibits lack of fit in places (discussed earlier). Finally, the current form of the model provides the potential for enhanced mechanistic insights and facilitates future hypothesis generation regarding the mechanisms of disease states. For example, insulin resistance involves IRS1 phosphorylation modulated by protein tyrosine phosphatase 1B (PTP1B)^29^ and modulated feedback between p70S6K and IRS1.^87^ In addition, the anabolic resistance of sarcopenia could involve any of the components in the signaling network, which our model is well positioned to explore.

## STAR★Methods

### Key resources table

**Table:** 

REAGENT or RESOURCE	SOURCE	IDENTIFIER
**Deposited data**		

Generated model, code, and data	This paper	https://doi.org/10.5281/zenodo.10205081
Model topology: mTOR signaling module	Dalle Pezze et al.^46^	https://doi.org/10.1126/scisignal.2002469
Model topology: leucine kinetic module	Tessari et al.^43^	https://doi.org/10.1152/ajpendo.1995.269.1.E127
Model topology: insulin secretion module	Sturis et al.^41^	https://doi.org/10.1152/ajpendo.1991.260.5.E801
Calibration: initial signaling protein concentrations	Wiśniewski et al.^88^	https://doi.org/10.1074/mcp.M113.037309
Calibration: initial signaling protein concentrations	Gonzalez-Franquesa et al.^89^	https://doi.org/10.1016/j.celrep.2021.109180
Calibration: time-course data	Glynn et al.^53^	https://doi.org/10.3945/jn.110.127647
Calibration: time-course data	Drummond et al.^52^	https://doi.org/10.1152/ajpendo.00690.2009
Validation: time-course data	Glynn et al.^53^	https://doi.org/10.3945/jn.110.127647
Validation: time-course data	Mitchell et al.^54^	https://doi.org/10.3945/jn.114.199604
Validation: time-course data	Wilkinson et al.^12^	https://doi.org/10.1113/jphysiol.2013.253203
Validation: time-course data	Drummond et al.^52^	https://doi.org/10.1152/ajpendo.00690.2009
Validation: time-course data	Dickinson et al.^55^	https://doi.org/10.3945/jn.111.139485

**Software and algorithms**		

MATLAB R2022a (9.12.0.2170939)	Mathworks	https://www.mathworks.com
CellDesigner 4.4.2	Systems Biology Institute^90^	https://www.celldesigner.org
R v4.3.1	R Core Team	https://www.r-project.org
R Studio v2023.06.1 + 524	Posit Software	https://posit.co/download/rstudio-desktop/
ggplot2	Wickham, 2016^91^	https://ggplot2.tidyverse.org
WebPlotDigitizer	Rohatgi, 2022^92^	https://automeris.io/WebPlotDigitizer/

### Resource availability

#### Lead contact

Further information and requests for resources and reagents should be directed to and will be fulfilled by the lead contact, David C. Clarke (dcclarke@sfu.ca).

#### Materials availability

This study did not generate new unique reagents.

#### Data and code availability

• This paper analyzes existing, publicly available data. These accession numbers for the datasets are listed in the key resources table. The curated data used for model calibration and validation is publicly available in the GitHub repository: https://doi.org/10.5281/zenodo.10205081.
• All original code has been deposited in the GitHub repository and is publicly available as of the date of publication: https://doi.org/10.5281/zenodo.10205081.
• Any additional information required to reanalyze the data reported in this paper is available from the lead contact upon request.

### Method details

#### System definition and simplifying assumptions

We defined our system to consist of four compartments: 1) stomach, 2) gut, 3) blood plasma/interstitial fluid, and 4) skeletal muscle. The system is stimulated through leucine feeding, in which fed leucine travels through a digestive system input module and is absorbed into the blood plasma compartment. The blood plasma compartment subsequently drives the cellular signaling and leucine dynamics in the skeletal muscle compartment. Each compartment is assumed to behave as a well-stirred tank reactor, and the modeled proteins were assumed to exist in sufficient concentrations to behave deterministically. These simplifying assumptions enabled us to use ordinary differential equations (ODEs) as the model’s mathematical framework.

#### Model topology and biochemistry considerations

Our model features four modules: 1) mTOR signaling module, 2) leucine kinetic module, 3) digestive system module, and 4) insulin secretion module.

##### The mTOR signaling module

The starting framework for the mTOR module was the model of Dalle Pezze et al.^46^ The Dalle Pezze et al.^46^ model simulates the insulin signaling dynamics propagating across the IR/Akt/mTORC1/p70S6K axis. The model also included an independent pathway for amino-acid-stimulated mTORC1 activity. We replicated the model representing their “hypothesis four” by using their ODEs, parameter values, and initial conditions. We were able to replicate their model outputs after slightly adjusting two of the parameter values. This result supports the correctness of the code upon which subsequent model iterations were developed.

The Dalle Pezze et al.^46^ model featured three shortcomings that we addressed to fulfill our modeling objectives. First, the model inputs (insulin and amino acids) were set as constant values for the simulation duration,^46^ which is inconsistent with the fact that both are dynamic, especially following feeding. Second, the model was developed using data from HeLa cells, such that we needed to adapt the model to human skeletal muscle. Third, the IRS1-PI3K module contained a degradation (“sink”) term. This term simplifies the IRS1-PI3K module, so we replaced the sink term with a more explicit representation of the signaling mechanisms. Specifically, we incorporated three IRS1 species (IRS1, phospho-IRS1^Y^, and phospho-IRS1^S^) and specified an association reaction between phospho-IRS1^Y^ and PI3K to form the phospho-IRS1^Y^-PI3K complex that promotes PDK1 phosphorylation. Several serine residues on IRS1 can be phosphorylated, which we designated phospho-IRS^S^ to collectively represent the species, because each inhibits downstream pathways in a similar manner.

##### The leucine kinetic module

We developed functions to simulate the dynamic amino acid and insulin inputs to the model. Leucine is the amino acid that most potently stimulates mTORC1 activity,^16^^,^^17^^,^^18^ such that we aimed to locate models of leucine kinetics that could replace the amino acid input from the Dalle Pezze et al.^46^ model. We located the Tessari et al.^43^ model of leucine kinetics, which used six compartments to simulate the dynamics of leucine across the human forearm. We developed a working model of leucine dynamics using the rate equations and flow rates provided in Tessari et al.,^43^ but the model needed to be modified in several ways to facilitate its integration with the mTOR signaling module. First, we assumed that the blood plasma/interstitial fluid compartment in our model was a well-mixed reactor (i.e., a mixture of arterial and venous blood) so we removed the venous leucine and KIC compartments. Removal of these compartments reduced the model from six to four compartments. The differences in concentrations between the arterial and venous compartments of leucine and KIC were 0.1 and 0.7 μmol/L, respectively, which we considered negligible (i.e., arterial leucine = 122.9 μmol/L, venous leucine = 122.8 μmol/L; arterial KIC = 25.8 μmol/L, venous KIC = 26.5 μmol/L).

Second, Tessari et al.^43^ reported transport rates rather than rate constants, such that we calculated the latter using the following equation:

(Equation 1) $$Kinetic\;rate\;parameter=Transport\;Rate\times Forearm\;Volume\times \frac{1}{100\;mL\;forearm\;volume}\times \frac{1}{moles\;of\;the\;previous\;species}$$

where the transport rate is in units of ${nmol\cdot \min}^{-1}\cdot {100\;mL}^{-1}$, forearm volume is in units of mL, and moles of the previous species is in nmol.

##### The digestive system module

We added a digestive system module that operates as an input function to simulate the dynamics of leucine ingestion and absorption in the blood. This module included two compartments: stomach and gut, and two degradation terms: excretion of leucine via the gut (i.e., feces) and first-pass splanchnic extraction. The parameters for this module reflect the gastric emptying and absorption rate of leucine when ingested in a low-volume, low-caloric solution in the post-absorptive state^93^ and were informed using the true ileal digestibility of leucine (∼90%)^94^ and the first-pass splanchnic extraction of amino acids in young adults (23–29%).^95^^,^^96^

##### The insulin secretion module

We used the previously validated model of Sturis et al.^41^ to simulate physiologically realistic insulin dynamics. Insulin is secreted from the pancreas in an oscillatory pattern with a period of approximately 120 min (i.e., ultradian).^41^ The Sturis et al.^41^ model mimics the ultradian oscillations of insulin secretion by featuring four negative feedback loops: 1) elevated glucose concentrations stimulate insulin secretion which reduces glucose production, 2) elevated glucose concentrations stimulate insulin secretion which promotes glucose utilization, 3) glucose inhibits further glucose production, and 4) glucose promotes further glucose utilization. The Sturis et al.^41^ model includes three compartments (plasma insulin, intercellular insulin, plasma glucose) and three variables that represent the delay between plasma insulin and its effect on hepatic glucose production.

We modified the Sturis et al.^41^ model in two ways to integrate it with our model. First, all glucose- or insulin-specific parameters (i.e., any parameters with units of mg or mU, respectively) were converted to units of moles using the molecular mass of glucose (180.156 g/mol) or the insulin unit conversion factor provided by Sturis et al.^41^ (1 mU insulin ≅ 6.67 pmol). Second, we set the post-absorptive glucose infusion rate in the Sturis et al.^41^ model to 75 mg/min to maintain post-absorptive plasma insulin concentrations at approximately 26 pmol/L (i.e., the concentration of plasma insulin from the calibration dataset).

##### Integration of modules

Having successfully replicated the mTOR signaling and insulin secretion modules and having formed working modules for leucine kinetics and the digestive system, we integrated the four modules through four links. First, intracellular leucine replaced the constant amino acid stimulus from the Dalle Pezze et al.^46^ model as the activating stimulus for mTORC1. Second, we added phospho-p70S6K^T389^ as a controller of intracellular leucine incorporation into skeletal muscle protein (i.e., protein synthesis) because phospho-p70S6K controls protein synthesis.^31^^,^^97^ Third, leucine is an insulin secretagogue,^23^ such that we added a link to simulate leucine-mediated insulin secretion. Lastly, insulin regulates skeletal muscle mass primarily by reducing MPB via interactions between Akt/FoxO3 and mTORC1/ULK1.^22^^,^^32^^,^^36^^,^^37^ Therefore, we inserted inhibitory links between phospho-Akt^T308^,^98^ and active mTORC1 to MPB, such that elevated phospho-Akt^T308^ and/or activated mTORC1 reduce MPB.

After integrating the modules, we expanded the model topology to represent the current state of knowledge in the literature while fostering model parsimony. We made four modifications to the model.

(1) Akt is an AGC kinase that must be phosphorylated twice for full activity.^46^ PDK1 and a PDK2 phosphorylate the Thr308 and Ser473 residues of Akt, respectively. mTORC2 is a *bona fide* PDK2 that phosphorylates the Ser473 residue of Akt.^46^ However, Dalle Pezze et al.^46^ were unable to reproduce experimental data for phospho-Akt^S473^ with mTORC2 alone, such that they introduced an additional PDK2 component to resolve the model output. However, the literature suggests that mTORC2 is the primary PDK2 that drives phospho-Akt^S473,^^99^^,^^100^ such that we felt justified in removing the additional PDK2 species.
(2) We expanded the Akt module to include all possible combinations of its phosphorylation states (i.e., Akt^T308^, Akt^S473^, Akt^S473,T308^).^28^
(3) Phospho-p70S6K inhibits mTORC1 activity through a negative feedback loop that results in the phosphorylation of mTORC1 at Ser2448, a residue that resides in the mTOR negative regulatory domain.^61^^,^^101^^,^^102^^,^^103^ However, the Dalle Pezze et al.^46^ model used phospho-mTORC1^S2448^ as a marker of mTORC1 activity. We therefore redefined the mTORC1 species as either *active* or *inactive* and included a negative feedback loop from phospho-p70S6K to mTORC1.
(4) For the sake of model parsimony, we removed the proline-rich Akt substrate of 40 kDa (PRAS40) because of a lack of experimental data for model fitting and because PRAS40 did not influence other model components.

A schematic diagram of the final model topology is presented in Figure 1. The model includes four defined compartments (stomach, gut, blood plasma, and skeletal muscle cells), 34 species (11 proteins, 13 post-translationally modified proteins, and 10 pools; Table S1), 64 kinetic parameters (61 adjustable, three constrained), and three delay parameters between insulin and glucose production as per the Sturis et al.^41^ model. All equations in our model were assumed to follow mass-action kinetics. The overall model consisted of 37 nonlinear ODEs that describe the rate of change of the number of moles of each molecular species within the indicated compartment (Tables S2 and S3). The number of moles of each species are determined by the sum of the reactions that generate and consume each species, which was expressed mathematically according to Equation 2.

(Equation 2) $$\frac{d}{dt}\left(species\right)=\sum {rates}_{generation}-\sum {rates}_{consumption}$$

All model runs were initiated with a burn-in period of 300 min to allow all model species to reach steady state. A plasma leucine infusion rate of 2.5 mg/min was used during the equilibrium period to maintain the physiological concentrations of species in the leucine module. Model code is available in the GitHub repository: https://doi.org/10.5281/zenodo.10205081.

#### Model calibration

Our model was calibrated similar to the procedure outlined in Zhao et al.^104^ First, we changed the initial conditions of the proteins to values representative of human skeletal muscle (Table S4). The concentration for plasma insulin was set to 28 p.m.^105^ The initial concentration for plasma leucine was set to 121 μM.^53^ The initial value for plasma α-ketoisocaproate (KIC) was set to 30.6 μM.^43^ The initial concentration for intracellular leucine was set to 128 μM.^52^ The initial concentration of total leucine bound to skeletal muscle protein was set to 134 mM using the approach in Wolfe et al.^94^ Skeletal muscle mass was assumed to constitute 40% body weight and approximately 20% of that mass is assumed to be muscle protein. Assuming an average body mass of 72.1 kg, then the assumed skeletal muscle protein mass would be ∼5.8 kg (72.1 kg × 40 % skeletal muscle × 20% muscle protein). We assumed that leucine comprises 8% of muscle protein mass,^44^ such that there is ∼460 g of leucine content in skeletal muscle. We were unable to find experimentally measured concentrations for intracellular KIC, so we set its initial value to 0 M, allowed the species to reach a steady-state value during the equilibration phase of the model simulations, and used the value that was reached at the end of the equilibration phase for all subsequent model simulations (11.5 μM).

We searched the literature to obtain plausible ranges for the initial concentrations of the insulin signaling proteins, but we were unable to locate their concentrations in human skeletal muscle cells. However, we found copy number values for most of the signaling proteins from quantitative proteomic studies of mouse skeletal muscle^88^^,^^89^ (Table S4). Skeletal muscle is multinucleated such that the copy number values in these studies were reported per skeletal muscle nucleus. To convert the copy number of signaling proteins per skeletal muscle nucleus to concentrations we used several literature-based estimates. These estimates included the number of nuclei per mouse skeletal muscle fiber (100–462 nuclei/cell^106^^,^^107^), the cellular protein content per volume (200–300 g protein per liter of cell volume^88^^,^^108^^,^^109^), and the total protein content per skeletal muscle cell nucleus (675 pg/nucleus in mouse leg skeletal muscle^88^; 2 ng/nucleus in mouse gastrocnemius^106^). We used these three values to estimate the volume of mouse leg skeletal muscle (Equation 3):

(Equation 3) $$cell\;volume=\frac{protein\;content\;per\;nucleus\times nuclei\;content}{cellular\;protein\;content\;per\;volume}$$

where volume is calculated in units of liters, proteincontentpernucleus is in units of grams per nucleus, nucleicontent is in units of nuclei per cell, and cellularproteincontentpervolume is in units of grams per liter. Using variations of the above estimates, we calculated the volume of mouse skeletal muscle fibers to be between 0.34 and 3.6 nL. With estimates for mouse skeletal muscle cell volume, we then converted the copy number values to concentrations (Equation 4):

(Equation 4) $${protein\;concentration}_{i}=\frac{{copy\;number}_{i}\times nuclei\;content}{N_{A}\times cell\;volume}$$

where the protein concentration of protein i is in units of mol/L, copy number of protein i is in units of copies per nucleus, $N_{A}$ is Avogadro’s constant, and volume is in units of liters. Of note, the number of nuclei per muscle fiber does not affect the protein concentration because the nuclei content is factored out in Equation 4. Protein concentrations are typically well conserved across cells,^110^ such that we assume that the calculated protein concentrations in mouse skeletal muscle are representative of the protein concentrations in human skeletal muscle. We set the initial concentrations of all phosphorylated proteins to 1% of the non-phosphorylated protein concentrations.^111^

Once the initial conditions of all proteins were calibrated to values representative of those in human skeletal muscle, we converted all concentrations to moles to allow us to simulate the rate of change of particles for each molecular species (i.e., we factored out the volume of the respective compartment). Converting species from concentrations to moles allowed us to ignore the influence of different compartment volumes within the model (i.e., movement of molecules across compartments of different volumes have disproportionate concentration changes). To convert species from concentrations to moles, we needed to estimate the volumes of each compartment. We used general subject characteristics from Caucasian males to calculate the compartment volumes muscle (age: 41.9 years old, mass: 72.1 kg, height: 1.73 m, resistance index (height^2^/Ω): 57.9 cm^2^/Ω).^112^ We calculated blood plasma volume using the Nadler formula for men^113^ (Equation 5):

(Equation 5) $$Blood\;Volume=\left(0.3669\times {Height}^{3}\right)+\left(0.03219\times Weight\right)+0.6041$$

where blood volume is given in liters and height and weight are in units of meters and kilograms, respectively. We estimated the skeletal muscle volume by first estimating total skeletal muscle mass using the regression equation developed by Janssen et al.^112^ (Equation 6):

(Equation 6) $$Skeletal\;Muscle\;Mass=\left[\left(\frac{{Height}^{2}}{R}\times 0.401\right)+\left(Gender\times 3.825\right)+\left(Age\times -0.071\right)\right]+5.102$$

where R is the bioelectrical impedance analysis resistance in Ohms, height is in centimeters, age is in years, and gender is given a value of 1 or 0 for men and women, respectively. We then used the skeletal muscle mass to calculate the volume of skeletal muscle using the density value for mammalian skeletal muscle (1.112 g/mL).^114^

Next, we adjusted the kinetic rate parameters (Table S5). To calibrate the kinetic rate parameters, we curated experimental time course data of eight readouts following insulin or leucine stimulation: plasma insulin,^53^ plasma leucine,^53^ intracellular leucine,^52^ phospho-IR,^115^ phospho-Akt^S473^, phospho-p70S6K1^T389^, and parameters from the three-pool model of leg amino acid kinetics [i.e., F_m,a_: inward amino acid transport from artery to muscle; F_m,0_: intracellular amino acid appearance from endogenous sources (i.e., proteolysis, *de novo* synthesis)].^53^ The phospho-Akt^S473^ and phospho-p70S6K1^T389^ time-courses were obtained by meta-analyzing studies that measured the respective protein in non-exercised, young adults following leucine ingestion (Figure S6, further details discussed below). Where necessary, we supplemented the muscle-specific data in response to leucine feeding with time courses following whey protein feeding (plasma KIC^56^) and from other cell types, including L6 myotubes (Akt^T308^,^116^), and 3T3-L1 adipocyte-like cells (IRS1^117^). We used these data to manually tune the parameter values to achieve a reasonable visual fit. We then used numerical optimization (“fmincon” and “GlobalSearch” functions in MATLAB) to fit the model parameters to quantitative time-courses of plasma insulin, plasma leucine, F_m,a_, and F_m,0_, to semi-quantitative time-courses (i.e., immunoblot data) of phospho-Akt^S473^ and phospho-p70S6K^T389^, and FSR data in response to a 3.5-gram bolus of leucine (Table S6). We used FSR data^53^ in the model calibration as a measure of the total MPS response (i.e., total grams of leucine incorporated into skeletal muscle over the intervention duration). We simulated the total MPS response in the model by measuring the AUC (“cumtrapz” function in MATLAB) of the MPS reaction (r15), we then converted the value from total moles over the intervention period to total grams using the leucine molar mass. We used the method in Wolfe et al.^94^ to convert experimentally measured FSR to total grams of leucine synthesized over the intervention period (Equation 7).

(Equation 7) $$Total\;leucine\;synthesized=Total\;leucine\;content\;bound\;to\;skeletal\;muscle\;protein\times \frac{\left({FSR}_{t_{1}}-{FSR}_{t_{0}}\right)}{100}\times t_{\exp}$$

where Totalleucinecontentboundtoskeletalmuscleprotein is calculated as previously described, ${FSR}_{t_{0}}$ and ${FSR}_{t_{1}}$ are the FSR values at the start and end of the experimental intervention, respectively, in units of %/hour, and $t_{\exp}$ is the experimental intervention duration in hours.

The phospho-protein data used in model calibration were measured using immunoblot analyses and were presented as fold changes from baseline, such that we converted the values to quantitative data that we could use in the model optimizer (Equation 8).

(Equation 8) $${\left[Protein\right]}_{i}={{Protein}_{\Delta }}_{i}\times \left[{Protein}_{t=0}\right]$$

where ${\left[Protein\right]}_{i}$ is the concentration of the protein at time i, ${{Protein}_{\Delta }}_{i}$ is the fold change of the protein at time i determined by the immunoblot analysis, and $\left[{Protein}_{t=0}\right]$ is the initial concentration of the protein.

The cost function for parameter optimization was the root-mean-square formula^87^^,^^104^^,^^118^ (Equation 9).

(Equation 9) $$V\left(p\right)=\frac{\sqrt{\sum_{i=1}^{N}\frac{{\left(y\left(i\right)-\hat{y}\left(i,p\right)\right)}^{2}}{{\sigma \left(i\right)}^{2}}}}{N}$$

where y(i) represents the ith experimental data point, $\hat{y}\left(i,p\right)$ is the model predicted value given parameter p, σ(i) is the standard deviation of the experimental data, and N is the number of experimental data points for that parameter. The index i includes all time points at which each protein was measured.

##### Meta-analysis of phospho-p70S6K^T389^ and phospho-Akt^S473^ data

Preliminary attempts to fit the model to the phospho-p70S6K^T389^ from Glynn et al. (increased leucine concentration group) resulted in unsatisfactory model fits. The inability of the model to fit the phospho-p70S6K^T389^ data prompted us to examine in detail the existing data regarding p70S6K phosphorylation following feeding. We searched in the PubMed database to locate studies that met the following eligibility criteria.

(1) Participants of the intervention group were healthy, young adults.
(2) The intervention provided a single bolus of leucine.
(3) Time-courses of skeletal muscle phospho-p70S6K^T389^ were reported (mean, standard error) following the ingestion of leucine.
(4) Participants were studied in the non-exercised state.

Our search string included the keywords p70S6K, leucine, human, and skeletal muscle but we were unable to find eligible articles. We revisited the studies that were identified during our data accession and we were able to locate several articles that met our eligibility criteria. We hand searched the publication history of the primary investigators from the eligible studies to locate additional studies. We extracted the data from each study (e.g., leucine dose, phospho-data fold change, standard error) and inputted the data into a spreadsheet. We then meta-analyzed the data using spline regression (‘gam’ function in R) to fit the extracted data and used the model to predict the time course of phospho-p70S6K at 0, 30, 60, 90, 120, 150, and 180 minutes (‘predict.gam’ function in R). The same protocol was applied to phospho-Akt^S473^.

Our systematic review of phospho-p70S6K^T389^ time-courses identified six eligible articles that featured seven independent intervention groups of young adults from three distinct research groups (Atherton,^12^^,^^54^ Moore,^60^^,^^119^ Rasmussen^53^^,^^120^). We found that the studies from the Rasmussen lab featured more discrepant fold changes in phospho-p70S6K^T389^ (3.85–39.5 fold change, Figure S6A) at time 60 minutes in comparison to the data from the other two groups. We were unable to discern from the methods the reasons for these discrepant results, so we removed these studies from further analysis. Following the removal of these studies, four eligible articles remained that featured four independent intervention groups. We meta-analyzed the data using spline regression to quantify the time course of phospho-p70S6K^T389^ fold changes, with leucine dose included as a covariate (k = 4, adjusted R^2^ = 0.64, deviance explained = 74%, n = 15; Figure S6B). We used the resulting model to predict the time-course of phospho-p70S6K^T389^ in response to a 3.5-gram leucine bolus for future model calibration.

The systematic review of phospho-Akt^S473^ time-courses located five eligible studies that featured five independent intervention groups.^12^^,^^53^^,^^54^^,^^119^^,^^120^ We applied spline regression to quantify the relationship of phospho-Akt^S473^ fold change over time in response to the ingestion of a bolus of leucine, in which leucine dose was included as a covariate (k = 5, adjusted R^2^ = 0.46, deviance explained = 58.1%, n = 22; Figure S6C). We used the resulting model to predict the time-course of phospho-Akt^S473^ in response to a 3.5-gram leucine bolus for future model calibration.

#### Model validation

We validated the calibrated model against six independent datasets with distinct feeding protocols: pulsatile feeding (i.e., four 0.9-gram boluses of leucine administered at 0, 45, 90, and 135 minutes^54^) and single boluses of leucine of different amounts (3.59 g^52^, 3.5 g^54^, 3.42 g^12^, 1.85 g^55^, and 1.8 g^53^; Table S7). We simulated the protocols by altering only the amount and timing of the ingested leucine to match the corresponding study protocols. No changes were made to any other model parameters or initial conditions in the model validation analyses.

### Quantification and statistical analysis

Root mean square errors are reported in each figure if corresponding experimental data were available. The total leucine synthesized over the intervention period is reported in each figure. Net muscle protein balance (“net balance”) was calculated as the difference between MPS and MPB (NB = MPS – MPB). Values of NB above zero indicate net synthesis while those below zero indicate net breakdown. Simulated phospho-Akt^S473^ dynamics in all main text and supplementary figures (excluding Figure S3) are plotted as the sum of the phospho-Akt^S473^ and phospho-Akt^S,T^ model species (i.e., the total serine phosphorylated Akt molecules). Experimental data plotted in simulation figures are presented as means ± standard error (SE).

CellDesigner 4.4.2^90^ was used to illustrate the model topology. WebPlotDigitizer^92^ was used to extract published data (i.e., means, SE) that was only reported in figures. R (version 4.2.1) was used to calculate the spline regression and to predict immunoblot-specific data. The R package ggplot2^91^ was used to visualize results. MATLAB version R2022a was used for all model simulations, estimation of parameters, analyses, and calculations. We used the MATLAB ‘ode23s’ function to numerically integrate the model using default tolerances.
