# Supplementary material for: Kinetic modeling of leucine-mediated signaling and protein metabolism in human skeletal muscle
Source: iScience. 2023 Dec 5;27(1):108634. doi: 10.1016/j.isci.2023.108634 (PMC10767222; doi:10.1016/j.isci.2023.108634)
Supplement: Document S1. Figures S1–S6 and Tables S1–S8 [file mmc1.pdf]

iScience, Volume *27*

Supplemental information

Kinetic modeling of leucine-mediated signaling and protein metabolism in human skeletal muscle

Taylor J. McColl and David C. Clarke

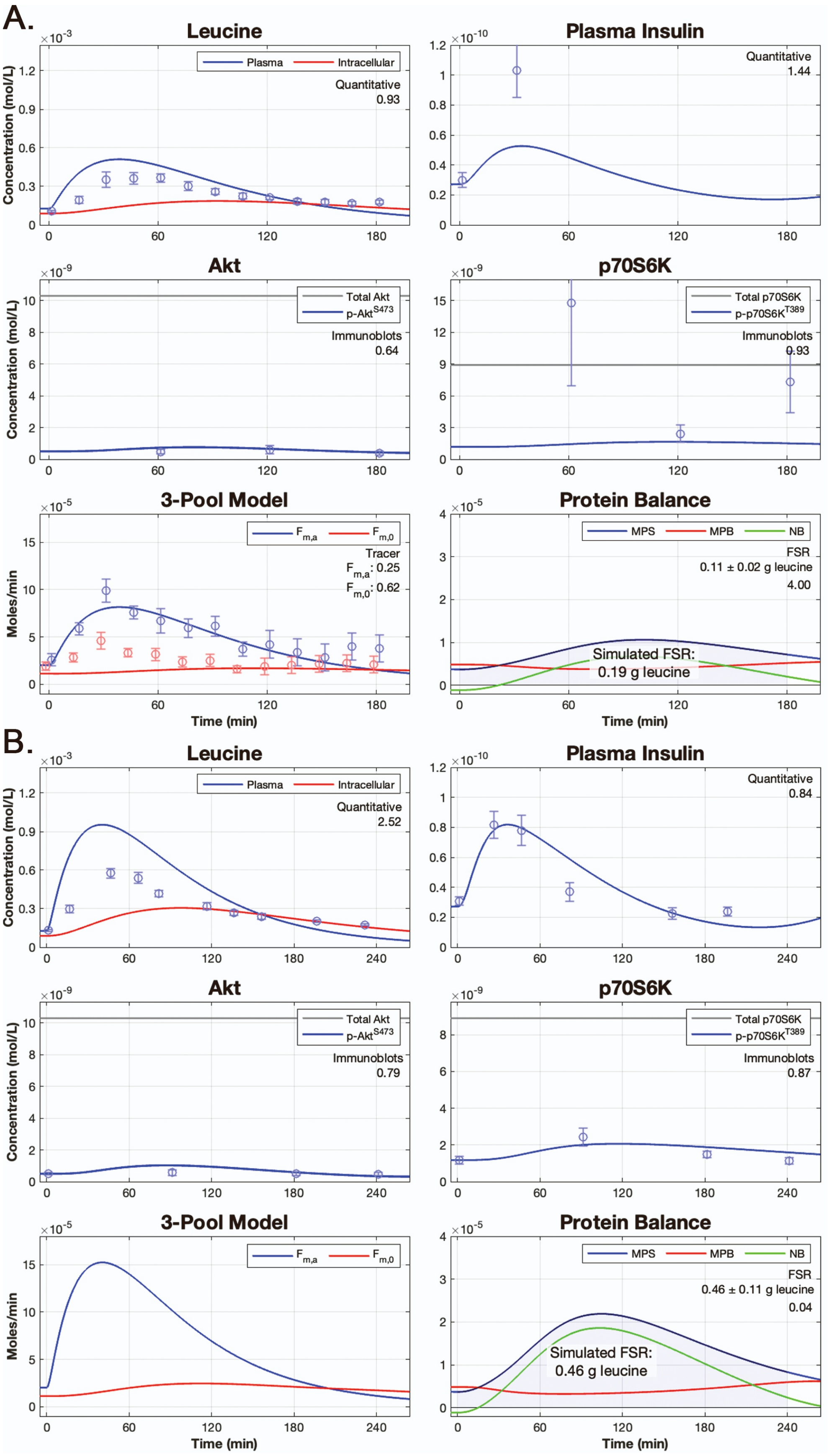

A.
Leucine
Plasma
Intracellular
Quantitative
0.93
Concentration (mol/L)
Plasma Insulin
Quantitative
1.44
Akt
Total Akt
p-Akt S473
Immunoblots
0.64
p70S6K
Total p70S6K
p-p70S6K T389
Immunoblots
0.93
3-Pool Model
Tracer
F m,a: 0.25
F m,0: 0.62
Moles/min
Time (min)
Protein Balance
MPS
MPB
NB
FSR
0.11 ± 0.02 g leucine
4.00
Simulated FSR:
0.19 g leucine
B.
Leucine
Quantitative
2.52
Plasma Insulin
Quantitative
0.84
Akt
Immunoblots
0.79
p70S6K
Immunoblots
0.87
3-Pool Model
Protein Balance
FSR
0.46 ± 0.11 g leucine
0.04
Simulated FSR:
0.46 g leucine

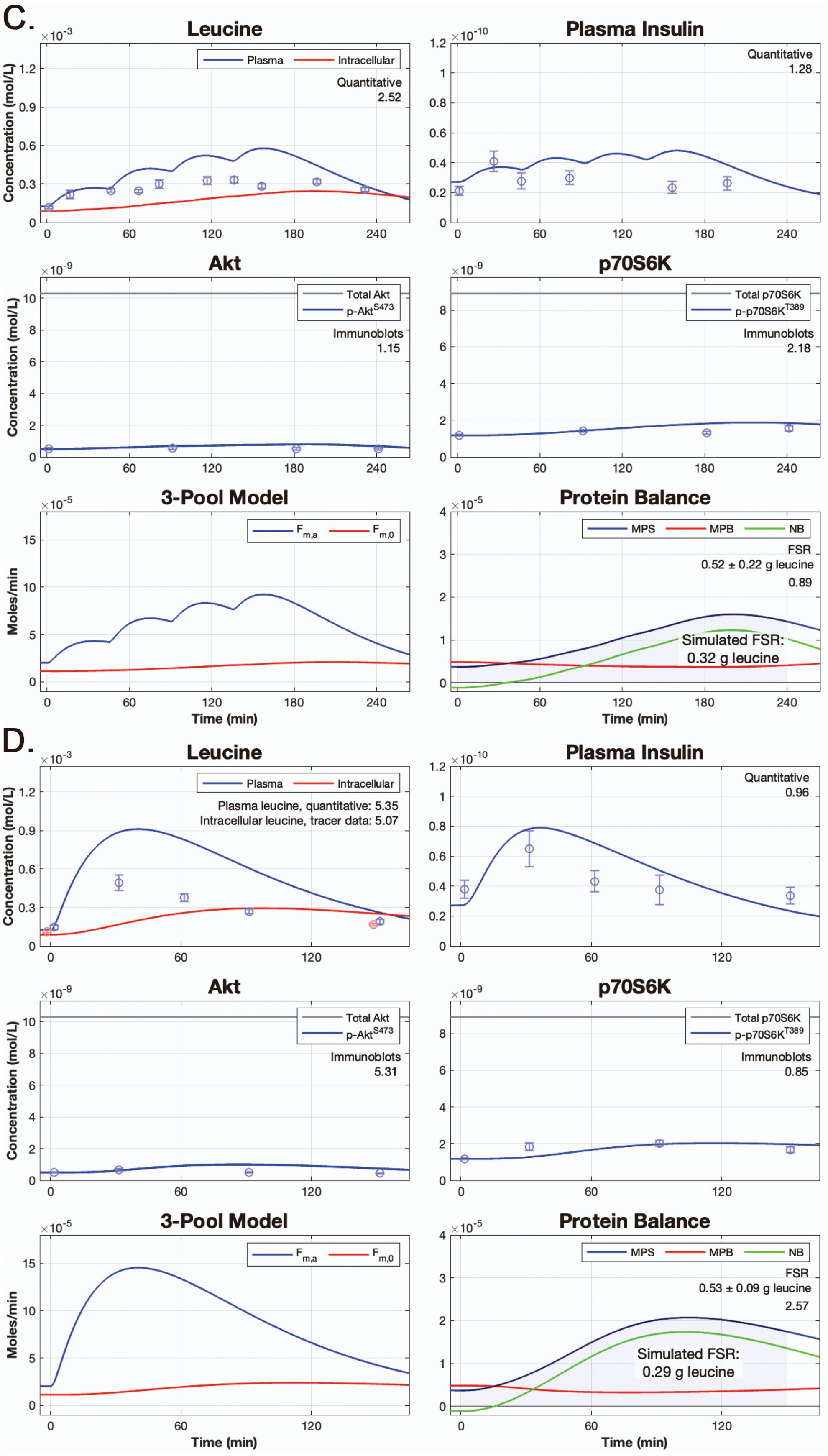
C.
Leucine
Plasma
Intracellular
Quantitative
2.52
Concentration (mol/L)
Plasma Insulin
Quantitative
1.28
Akt
Total Akt
p-Akt S473
Immunoblots
1.15
p70S6K
Total p70S6K
p-p70S6K T389
Immunoblots
2.18
3-Pool Model
F m,a
F m,0
Moles/min
Time (min)
Protein Balance
MPS
MPB
NB
FSR
0.52 ± 0.22 g leucine
0.89
Simulated FSR:
0.32 g leucine
D.
Leucine
Plasma
Intracellular
Plasma leucine, quantitative: 5.35
Intracellular leucine, tracer data: 5.07
Plasma Insulin
Quantitative
0.96
Akt
Total Akt
p-Akt S473
Immunoblots
5.31
p70S6K
Total p70S6K
p-p70S6K T389
Immunoblots
0.85
3-Pool Model
F m,a
F m,0
Protein Balance
MPS
MPB
NB
FSR
0.53 ± 0.09 g leucine
2.57
Simulated FSR:
0.29 g leucine
Time (min)

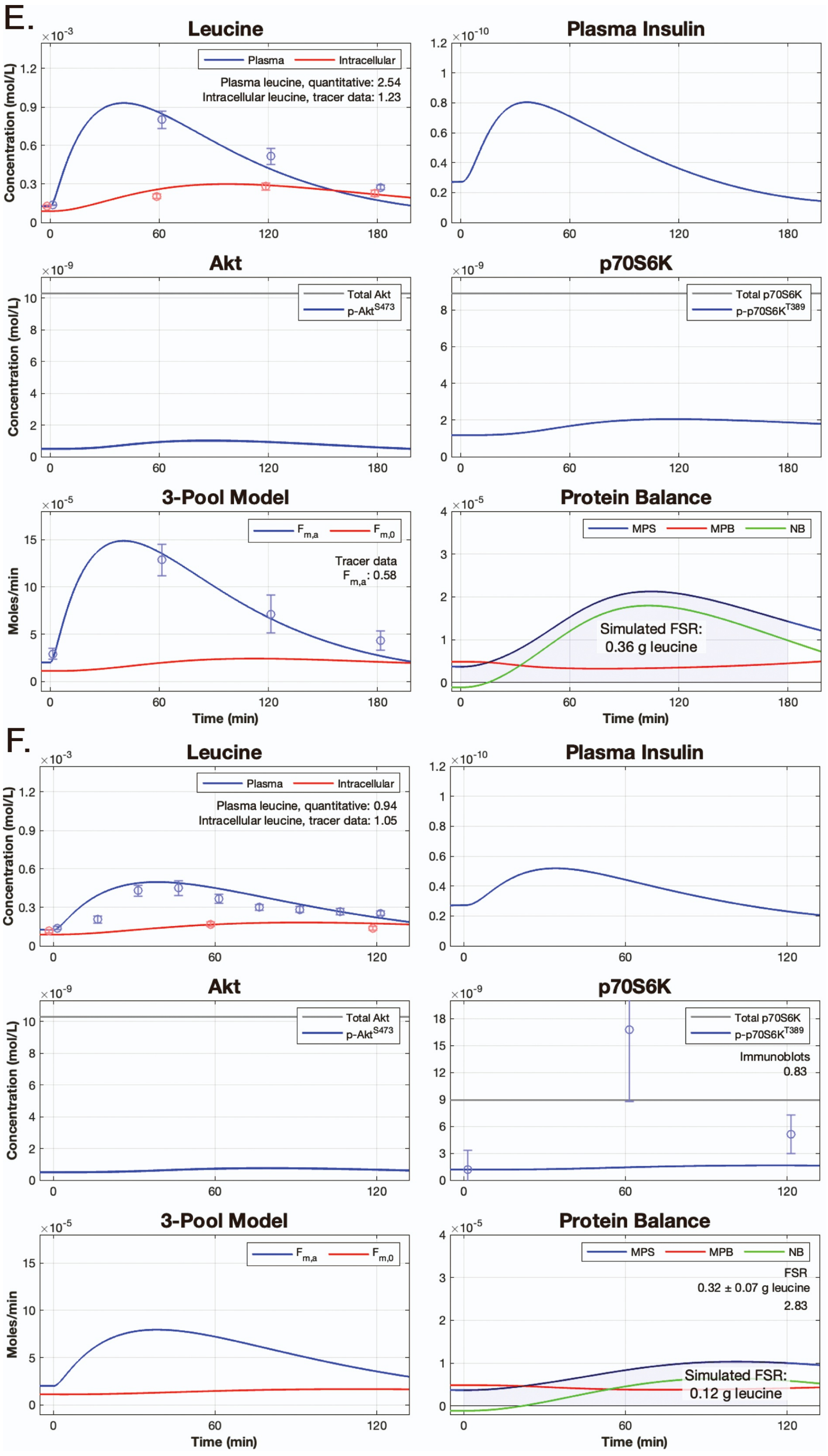
E.
Leucine
Plasma
Intracellular
Plasma leucine, quantitative: 2.54
Intracellular leucine, tracer data: 1.23
Plasma Insulin
Akt
Total Akt
p-Akt S473
p70S6K
Total p70S6K
p-p70S6K T389
3-Pool Model
Tracer data
F m,a: 0.58
Protein Balance
MPS
MPB
NB
Simulated FSR:
0.36 g leucine
Concentration (mol/L)
Moles/min
Time (min)
F.
Leucine
Plasma leucine, quantitative: 0.94
Intracellular leucine, tracer data: 1.05
Plasma Insulin
Akt
p70S6K
Immunoblots
0.83
3-Pool Model
Protein Balance
FSR
0.32 ± 0.07 g leucine
2.83
Simulated FSR:
0.12 g leucine

Figure S1. Comprehensive model validation. Related to Figure 2.

Simulated time-courses of plasma leucine, plasma insulin, Akt, p70S6K, three-pool model parameters $F_{m,a}$ and $F_{m,0}$, and muscle protein balance following a single (A) 1.85-gram, (B) 3.59-gram, (D) 3.42-gram, (E) 3.50-gram, or (F) 1.80-gram bolus of leucine or (C) pulsatile leucine feedings (0.59-grams of leucine provided at 0, 45, 90, and 135 minutes). Data points represent experimental data collected from (A) Glynn et al.[S1], (B,C) Mitchell et al.[S2], (D) Wilkinson et al.[S3], (E) Drummond et al.[S4], and (F) Dickinson et al[S5]. Root mean square values for each time-course with corresponding experimental data are included within the respective plot. The measured data are presented as means ± SE. FSR = fractional synthetic rate, MPS = muscle protein synthesis, MPB = muscle protein breakdown, NB = net balance.

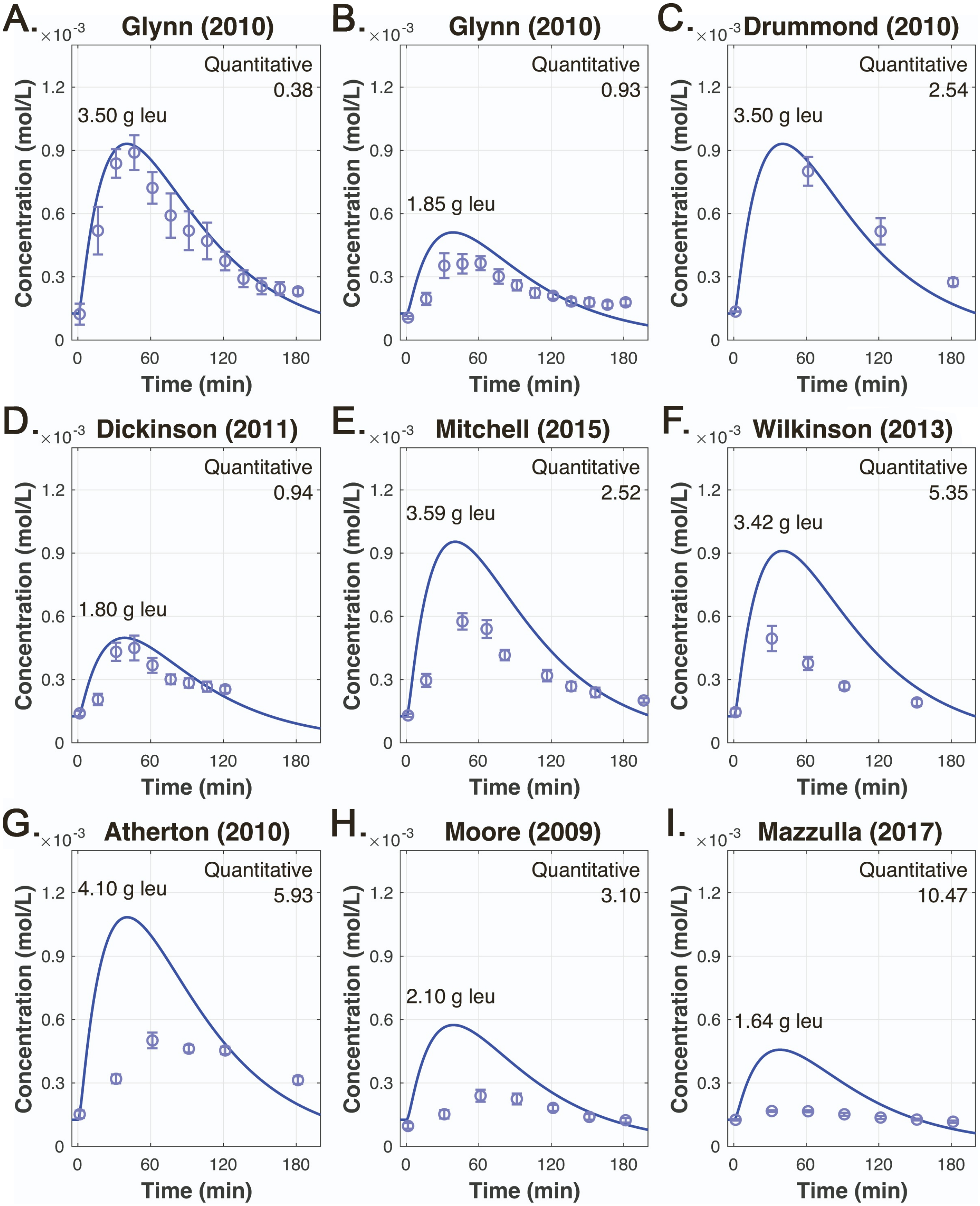

Figure S2. Discrepant plasma leucine measurements between different data sets. Related to Figure 2.

Simulated plasma leucine time-courses in response to bolus leucine feedings (A-F: 3.5 g, 1.85 g, 3.50 g, 1.80 g, 3.59 g, and 3.42 g, respectively) and whey protein feedings with differing leucine amounts (G-I: 4.1 g, 2.1 g, or 1.67 g, respectively). Data points represent experimental data from (A) Glynn et al.[S1], (B)

Glynn et al.[S1], (C) Drummond et al.[S4], (D) Dickinson et al.[S5], (E) Mitchell et al.[S2], (F) Wilkinson et al.[S3], (G) Atherton et al.[S6], (H) Moore et al.[S7], and (I) Mazzulla et al.[S8]. The ingested dose of leucine is listed on the left side of each plot and the root mean square values for each time-course is listed in the upper-right portion of each plot. The measured data are presented as means ± SE.

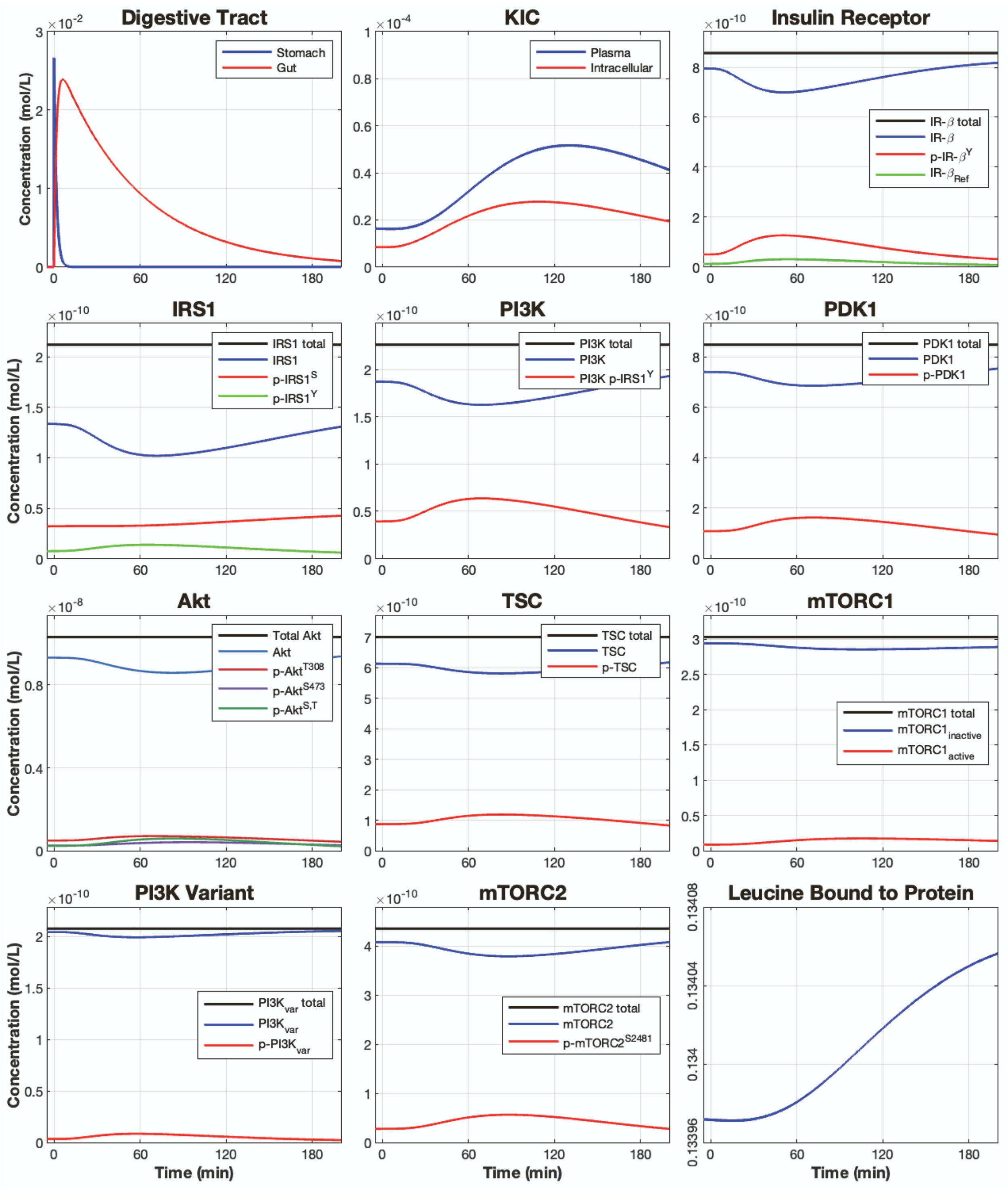

Figure S3. Simulation of unmeasured species in the model. Related to Figure 2.

Simulated time-courses of all model species that were not experimentally measured following a 3.5-gram bolus of leucine. Each of the four Akt species are plotted in the Akt plot (i.e., phospho-Akt^{S473} only represents the singular serine phosphorylated species). Total IRS1 includes the PI3K phospho-IRS1^{Y} complex, which is presented in the adjacent PI3K panel.

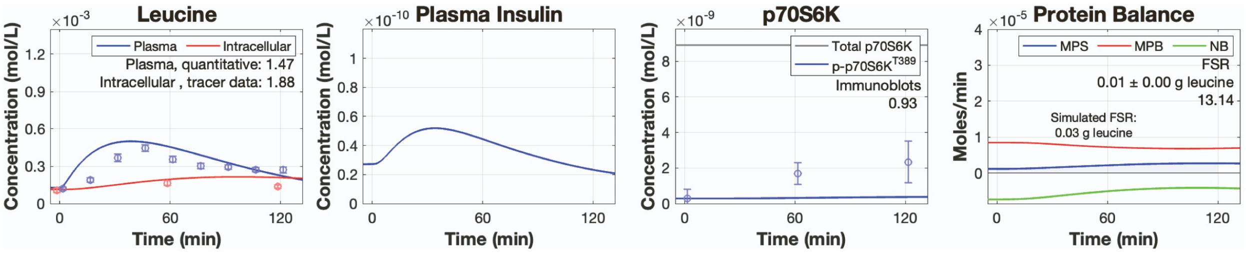

Figure S4. Model simulation of rapamycin ingestion. Related to Figure 3.
Simulated time-courses of plasma leucine, intracellular leucine, plasma insulin, p70S6K, and muscle protein balance following a 1.8-gram bolus of leucine with prior ingestion of rapamycin, a potent mTORC1 inhibitor. Data points represent experimental data from Dickinson et al.[S5]. Root mean square values for each time-course with corresponding experimental data are included within each plot. The measured data are presented as means ± SE. FSR = fractional synthetic rate, MPS = muscle protein synthesis, MPB = muscle protein breakdown, NB = net balance.

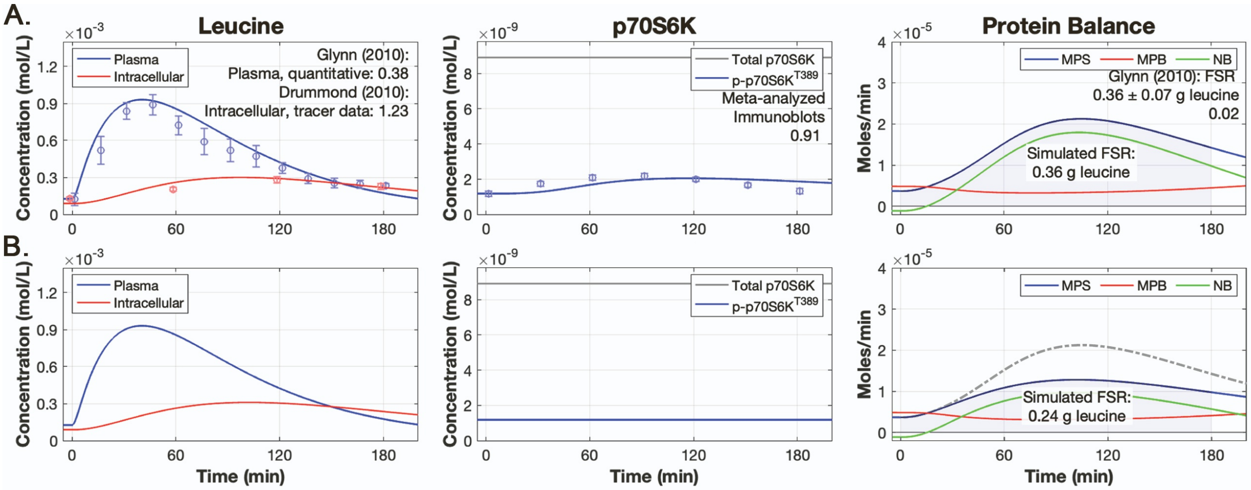

Figure S5. Phospho-p70S6K signaling is required to elicit a maximal MPS response. Related to Figure 3.

Simulated plasma leucine, intracellular leucine, p70S6K, and muscle protein balance time courses following a 3.5-gram bolus of leucine with (A) phospho-p70S6K sensitive to leucine-mediated signaling or (B) phospho-p70S6K insensitive to leucine-mediated signaling and maintained at the calibrated concentration over the simulation duration. Data points in (A) represent data from the model calibration[S1]. The gray dashed line in the (B) protein balance plot is a reference line for the original MPS from (A). Root mean square values for each time-course with corresponding experimental data are included within each plot. The measured data are presented as means ± SE. FSR = fractional synthetic rate, MPS = muscle protein synthesis, MPB = muscle protein breakdown, NB = net balance.

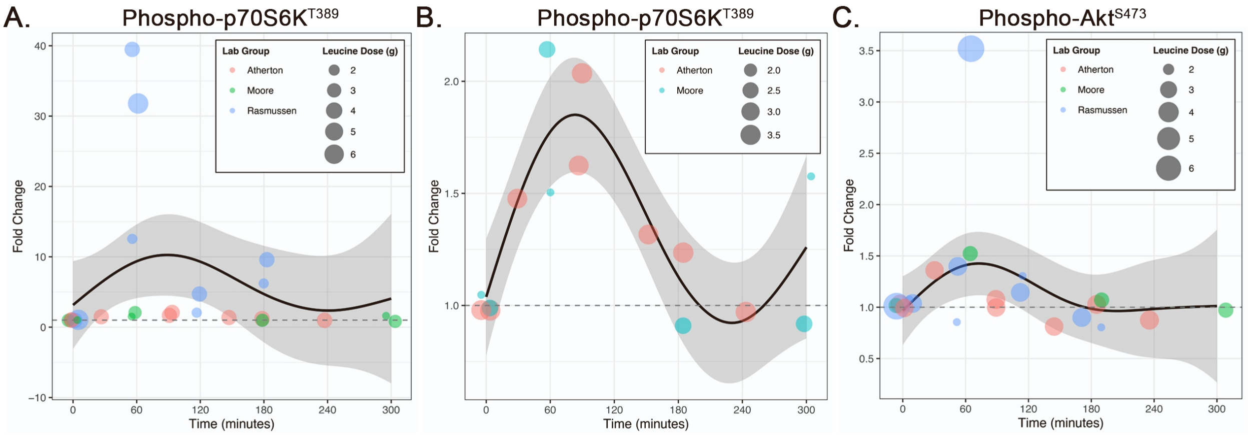

Figure S6. Meta-analysis of phospho-p70S6K^{T389} and phospho-Akt^{S473} time-course measurement in young adult skeletal muscle following leucine ingestion. Related to Figure 2.

Scatterplots of (A,B) phospho-p70S6K^{T389} or (C) phospho-Akt^{S473} fold changes versus time using the data extracted from the systematic review. The phospho-p70S6K^{T389} scatterplots either (A) include or (B) omit the data from the Rasmussen lab group. The black line is the predicted phospho-p70S6K^{T389} or phospho-Akt^{S473} time-course in response to a 3.5-gram bolus of leucine and the gray areas represent the 95% confidence error of the predicted phospho-protein time-course. The size of the data points corresponds to the leucine dose administered in the intervention. The color of data points corresponds to the lab group from which the data was reported. Data points are jittered to minimize overlap.

Table S1. Proteins included in the model and their properties. Related to Figure 2.

Species	Name	Properties	Ref.
Akt	Protein kinase B	Akt is an AGC kinase that must be phosphorylated twice to achieve full activity. Akt translocates to the plasma membrane by interacting with PIP_3, where PDK1 phosphorylates the Thr308 residue of Akt and mTORC2 phosphorylates the Ser473 residue of Akt. Thr308 and Ser473 may be independently phosphorylated such that there are two pathways that can achieve full activation of Akt. Akt^{T308} and $Akt^{T308,S473}$ function to phosphorylate the TSC1/2 complex.	S9-13
IR_{β}	Insulin receptor beta	Insulin binds to the IR, which leads to the phosphorylation of the Tyr1164 site and other tyrosine residues. Tyrosine phosphorylated IR promotes phosphorylation of the IRS1 tyrosine residues.	S10,S14
IRS1	Insulin receptor substrate 1	IRS1 can be phosphorylated at two sites: Ser636 and Tyr residue. The Tyr residue of IRS1 is phosphorylated by IR_b^{Y1164} and functions to activate the PI3K species. The Ser636 species is phosphorylated by $p70S6K^{T389}$, which disrupts the ability of IRS1 to interact with activated IR_b. Phosphorylation of the Ser636 residue denotes a negative feedback loop mediated by p70S6K that inhibits insulin-mediated mTORC1 activity.	S10,S14-18
mTORC1	Mechanistic target of rapamycin complex 1	mTORC1 activity is mediated through insulin- and leucine-independent pathways. Insulin activates mTORC1 through a signalling cascade that promotes TSC1/2 phosphorylation to inactivate TSC1/2 inhibition. Removal of TSC inhibition allows mTORC1 to translocate to the lysosome where it is activated by Rheb. Leucine activates the Ragulator-Rag complex that directs mTORC1 to the surface of lysosome where it is activated by Rheb. mTORC1 mediates protein translational dynamics by phosphorylating downstream proteins (e.g., p70S6K1, 4EBP1). mTORC1 is inhibited by PRAS40, which interacts with RAPTOR to negatively regulate kinase activity. Additionally, p70S6K regulates mTORC1 activity via a negative feedback loop in which it phosphorylates the Ser2448 residue.	S5,S10, S19-25
mTORC2	Mechanistic target of rapamycin complex 2	mTORC2 resides at the plasma membrane, is regulated by growth factors, and phosphorylates the Ser473 residue of Akt. In response to growth factors,	S10,S17, S26

		mTORC2 is phosphorylated at the Ser2481 residue, which promotes phosphorylation of the Akt Ser473 residue.	
p70S6K	p70 ribosomal protein S6 kinase	p70S6K1 is a serine/threonine kinase whose maximum activation requires phosphorylation of the Thr389 and Thr229 residues by mTORC1 and PDK1, respectively. p70S6K1 promotes translational control primarily by phosphorylating the proteins eukaryotic initiation factor (eIF) 4B, eIF3, and PDCD4 (programmed cell death protein 4). Additionally, p70S6K1 controls protein elongation by phosphorylating eukaryotic elongation factor (eEF) 2K and eEF1A [27]. Like p70S6K1, 4EBP1 controls translation by controlling initiation, ribosomal biogenesis, and export of mRNA from the nucleus. p70S6K participates in several feedback loops that function to reduce mTORC1 activity (e.g., p70S6K/IRS1, p70S6K/mTORC1).	S5,S10, S16,S20, S22-23, S27-30
PDK1	3-phosphoinositide dependent kinase-1	PDK1 is activated by PI3K and functions to phosphorylate the Thr308 residue of Akt and the Thr229 residue of p70S6K.	S9-10, S28,S30-31
PI3K	Phosphoinositide 3-kinase	Tyrosine phosphorylated IRS1 serves as a docking site for the p85 regulatory subunit of PI3K, which results in the activation of PI3K. Activated PI3K phosphorylates PI(4,5)P2 to form PIP3 that activates PDK1.	S9-10, S14,S32-33
$PI3K_{variant}$	PI3K variant	A wortmannin-sensitive, but IRS1 independent PI3K species proposed by Dalle Pezze et al. that is activated by IR_b and stimulates mTORC2 in response to insulin.	S12
TSC1/2	Tuberous sclerosis protein 1/2	The TSC1/2 complex negatively regulates mTORC1 activity. The complex is phosphorylated by Akt^{T308} and $Akt^{T308,S473}$, which inactivates the inhibitory function of the complex. This inactivation of the inhibitory function promotes the RHEB GTPase to form the active RHEB/GTP complex, which activates mTORC1.	S10,S12, S34-35

Table S2. Reaction rate equations. Related to Figure 2.
Reaction rate equations and the functional relationships used in the oscillatory insulin module.

Description of reaction	Reaction	Reaction rates
	Digestive module	
Leucine transport from stomach to gut	Stomach → Gut	$r1 = k1 \times Leucine_{stomach}$
Leucine excretion via the gut	Gut → ∅	$r2 = k2 \times Leucine_{gut}$
First pass splanchnic extraction	Gut → ∅	$r68 = k68 \times Leucine_{gut}$
Absorption of leucine from the gut to the blood plasma	Gut → Plasma leucine	$r3 = k3 \times Leucine_{gut}$
	Blood Plasma	
Leucine mediated insulin secretion	Plasma leucine → Plasma insulin	$r4 = k4 \times Leucine_{plasma}$
	Leucine kinetic module	
Leucine transport into skeletal muscle cells	Plasma leucine → Intracellular leucine	$r6 = k6 \times Leucine_{plasma}$
Leucine release from skeletal muscle to blood plasma	Intracellular leucine → Plasma leucine	$r7 = k7 \times (Leucine_{intracellular} - Leucine_{plasma})$
Protein synthesis	Intracellular leucine → Protein	$r15 = k15 \times Leucine_{intracellular} \times p70S6K^{T389}$
Akt-mediated protein breakdown (i.e., Akt/FoxO interaction)	Protein → Intracellular leucine	$r9 = k9 \times Protein \times \frac{1}{p\text{-}Akt^{T308}}$
mTORC1-mediated protein breakdown (i.e., mTORC1/ULK1 interaction)	Protein → Intracellular leucine	$r69 = k69 \times Protein \times \frac{1}{mTORC1_{active}}$
Leucine transamination to KIC	Intracellular leucine → Intracellular KIC	$r10 = k10 \times Leucine_{intracellular}$
KIC reamination to leucine	Intracellular KIC → Intracellular leucine	$r11 = k11 \times KIC_{intracellular}$
KIC transport into skeletal muscle cells	Plasma KIC → Intracellular KIC	$r12 = k12 \times KIC_{Plasma}$

KIC release from skeletal muscle to blood plasma	Intracellular KIC → Plasma KIC	$r13 = k13 \times KIC_{intracellular}$
KIC oxidation	Intracellular KIC → ∅	$r14 = k14 \times KIC_{intracellular}$
mTOR signalling module		
Insulin-mediated IR-β phosphorylation	IR-β → p-IR-β^{Y1164}	$r16 = k16 \times IR\text{-}\beta \times Insulin_{plasma}$
Dephosphorylation of p-IR-β^{Y1164}, refractory state	p-IR-β^{Y1164}→ IR-$\beta_{refractory}$	$r17 = k17 \times p\text{-}IR\text{-}\beta^{Y1164}$
Dephosphorylation of p-IR-β^{Y1164}, non-refractory state	IR-$\beta_{refractory}$ → IR-β	$r18 = k18 \times IR\text{-}\beta_{refractory}$
Phosphorylation of the IRS1 tyrosine site	IRS1 → p-IRS1^{Y}	$r19 = k19 \times IRS1 \times p\text{-}IR\text{-}\beta^{Y1164}$
Dephosphorylation of p-IRS1^{Y}	p-IRS1^{Y} → IRS1	$r20 = k20 \times p\text{-}IRS1^{Y}$
Phosphorylation of the IRS1 serine site	IRS1 → p-IRS1^{S}	$r21 = k21 \times IRS1 \times p\text{-}p70S6K1^{T389}$
Dephosphorylation of the p-IRS1^{S}	p-IRS1^{S} → IRS1	$r22 = k22 \times p\text{-}IRS1^{S}$
Docking of PI3K with p-IRS1^{Y}	p-IRS1^{Y} + PI3K → p-IRS1^{Y}/PI3K$_{clx}$	$r23 = k23 \times p\text{-}IRS1^{Y} \times PI3K$
Removal of PI3K from p-IRS1^{Y}	p-IRS1^{Y}/PI3K$_{clx}$ → p-IRS1^{Y} + PI3K	$r24 = k24 \times p\text{-}IRS1^{Y}\_PI3K_{clx}$
Phosphorylation of PDK1	PDK1 → p-PDK1	$r25 = k25 \times PDK1 \times p\text{-}IRS1^{Y}\_PI3K_{clx}$
Dephosphorylation of p-PDK1	p-PDK1 → PDK1	$r26 = k26 \times p\text{-}PDK1$
Phosphorylation of Akt at the T308 residue	Akt → p-Akt^{T308}	$r27 = k27 \times Akt \times p\text{-}PDK1$
Dephosphorylation of p-Akt^{T308}	p-Akt^{T308} → Akt	$r28 = k28 \times p\text{-}Akt^{T308}$
Phosphorylation of Akt at the S473 residue	Akt → p-Akt^{S473}	$r29 = k29 \times Akt \times p\text{-}mTORC2^{S2481}$
Dephosphorylation of p-Akt^{S473}	p-Akt^{S473} → Akt	$r30 = k30 \times p\text{-}Akt^{S473}$
Phosphorylation of Akt at the S473 residue to form the dual-phosphorylated species	p-Akt^{T308} → p-Akt^{T308,S473}	$r31 = k31 \times p\text{-}Akt^{T308} \times p\text{-}mTORC2^{S2481}$

Dephosphorylation of the serine residue from p-Akt$^{T308, S473}$	p-Akt^{T308,S473} → p-Akt^{T308}	$r32 = k32 \times p\text{-}Akt^{T308,S473}$
Phosphorylation of Akt at the T308 residue to form the dual-phosphorylated species	p-Akt^{S473} → p-Akt^{T308,S473}	$r33 = k33 \times Akt^{S473} \times p\text{-}PDK1$
Dephosphorylation of the threonine residue from p-Akt$^{T308, S473}$	p-Akt^{T308,S473} → p-Akt^{S473}	$r34 = k34 \times p\text{-}Akt^{T308,S473}$
Phosphorylation of the TSC complex via p-Akt$^{T308, S473}$	TSC_{clx} → p-TSC_{clx}	$r35 = k35 \times TSC_{clx} \times p\text{-}Akt^{T308,S473}$
Phosphorylation of the TSC complex via p-Akt^{T308}	TSC_{clx} → p-TSC_{clx}	$r36 = k36 \times TSC_{clx} \times p\text{-}Akt^{T308}$
Dephosphorylation of p-TSC complex	p-TSC_{clx} → TSC_{clx}	$r37 = k37 \times p\text{-}TSC_{clx}$
Inactivation of mTORC1 via TSC	$mTORC1_{active}$ → $mTORC1_{inactive}$	$r38 = k38 \times mTORC1_{active} \times TSC_{clx}$
Activation of mTORC1 via leucine stimulation	$mTORC1_{inactive}$ → $mTORC1_{active}$	$r39 = k39 \times mTORC1_{inactive} \times Leucine_{intracellular}$
Phosphorylation of p70S6K1 via mTORC1	p70S6K1 → p-p70S6K1^{T389}	$r40 = k40 \times p70S6K1 \times mTORC1_{active}$
Phosphorylation of p70S6K1 via p-PDK1	p70S6K1 → p-p70S6K1^{T389}	$r41 = k41 \times p70S6K1 \times p\text{-}PDK1$
Dephosphorylation of p-p70S6K1^{T389}	p-p70S6K1^{T389} → p70S6K1	$r42 = k42 \times p\text{-}p70S6K1^{T389}$
Inactivation of mTORC1 via p-p70S6K1^{T389}	$mTORC1_{active}$ → $mTORC1_{inactive}$	$r43 = k43 \times mTORC1_{active} \times p\text{-}p70S6K1^{T389}$
Phosphorylation of PI3K variant	$PI3K_{variant}$ → p-$PI3K_{variant}$	$r44 = k44 \times PI3K_{variant} \times p\text{-}IR\text{-}\beta^{Y1164}$
Dephosphorylation of p-PI3K variant	p-$PI3K_{variant}$ → $PI3K_{variant}$	$r45 = k45 \times p\text{-}PI3K_{variant}$
Phosphorylation of mTORC2	mTORC2 → p-mTORC2^{S2481}	$r46 = k46 \times mTORC2 \times p\text{-}PI3K_{variant}$

Dephosphorylation of mTORC2	p-mTORC2^{S2481} → mTORC2^{S2481}	$r47 = k47 \times p\text{-}mTORC2^{S2481}$
*Insulin oscillatory module (Sturis et al.[S37])**		
Effect of glucose on insulin secretion		$f_1(Glucose_{plasma}) = \frac{R_m}{1 + \exp\left(\frac{C_1 - \frac{Glucose_{plasma}}{V_g}}{a_1}\right)}$
Effect of insulin on glucose production		$f_2(Glucose_{plasma}) = U_b \cdot \left(1 - \exp\left(\frac{-Glucose_{plasma}}{C_2 \cdot V_g}\right)\right)$
Insulin independent glucose utilization		$f_3(Glucose_{plasma}) = \frac{Glucose_{plasma}}{C_3 \cdot V_g}$
Insulin dependent glucose utilization		$f_4(Insulin_{interstitial}) = U_o + \frac{U_m - U_o}{1 + exp\left(-\beta \cdot ln\left(\frac{Insulin_{interstitial}}{C_4} \cdot \left(\frac{1}{V_i} + \frac{1}{E \cdot t_i}\right)\right)\right)}$
Insulin dependent hepatic glucose production		$f_5(x_3) = \frac{R_g}{1 + \exp\left(\alpha \cdot \left(\frac{x_3}{V_p - C_5}\right)\right)}$

*The insulin oscillatory module reaction equations are as presented for the 'Original Insulin-Glucose Feedback Model' in Tolic et al.[S36]. These equations are simplified versions of the original equations presented in Sturis et al.[S37], such that they were more straightforward to replicate in our model.

Table S3. System of ordinary differential equations. Related to Figure 2.

Variable	Differential equations (with respect to time)
Leucine in the stomach	dx(1) = -r1
Leucine in the gut	dx(2) = r1 – r2 – r3 – r68
Plasma insulin	dx(3) = $f_1(Glucose_{plasma}) - E \cdot \left(\frac{Insulin_{plasma}}{V_p} - \frac{Insulin_{interstitial}}{V_i}\right) - \frac{Insulin_{plasma}}{t_p}$
Plasma leucine	Burn-in: dx(4) = r3 + r7 – r6 + $\text{Leucine}_{\text{infusion}}$<br>Simulation: dx(4) = r3 + r7 – r6
Plasma KIC	dx(5) = r13 – r12
Intracellular leucine	dx(6) = r6 + r9 + r11 + r69 – r7 – r10 – r15
Intracellular KIC	dx(7) = r10 + r12 – r11 – r13 – r14
Protein	dx(8) = r15 – r9 – r69
IR-β	dx(9) = r18 – r16
p-IR-β^{Y1164}	dx(10) = r16 – r17
p-IR-$\beta_{\text{refractory}}$	dx(11) = r17 – r18
IRS1	dx(12) = r20 + r22 – r19 – r21
p-IRS1^{Y}	dx(13) = r19 + r24 – r20 – r23
p-IRS1^{S}	dx(14) = r21 – r22
PI3K	dx(15) = r24 – r23
p-IRS1^{Y}_PI3K$_{\text{clx}}$	dx(16) = r23 – r24
PDK1	dx(17) = r26 – r25
p-PDK1	dx(18) = r25 – r26
Akt	dx(19) = r28 + r30 – r27 – r29
p-Akt^{T308}	dx(20) = r27 + r32 – r28 – r31
p-Akt^{S473}	dx(21) = r29 + r34 – r30 – r33
p-Akt^{T308,S473}	dx(22) = r31 + r33 – r32 – r34
TSC$_{\text{clx}}$	dx(23) = r37 – r35 – r36

p-TSC_{clx}	dx(24) = r35 + r36 – r37
$mTORC1_{inactive}$	dx(25) = r38 + r43 – r39
$mTORC1_{active}$	dx(26) = r39 – r38 – r43
p70S6K1	dx(27) = r42 – r40 – r41
p-p70S6K1^{T389}	dx(28) = r40 + r41 – r42
$PI3K_{variant}$	dx(29) = r45 – r44
p-$PI3K_{variant}$	dx(30) = r44 – r45
mTORC2	dx(31) = r47 – r46
p-mTORC2^{S2481}	dx(32) = r46 – r47
Intercellular insulin	dx(35) = $E \cdot \left(\frac{Insulin_{plasma}}{V_p} - \frac{Insulin_{interstitial}}{V_i}\right) - \frac{Insulin_{interstitial}}{t_i}$
Plasma glucose	dx(36) = $Glucose_{infusion} - f_2(Glucose_{plasma}) - f_3(Glucose_{plasma}) \cdot f_3(Insulin_{interstitial}) + f_5(x_3)$
x1	dx(37) = $\frac{3}{t_d} \cdot (Insulin_{insterstitial} - x_1)$
x2	dx(38) = $\frac{3}{t_d} \cdot (x_1 - x_2)$
x3	dx(39) = $\frac{3}{t_d} \cdot (x_2 - x_3)$

Table S4. Model species and initial concentrations. Related to Figure 2.
Model species (state variables) and their initial concentrations before and after the model calibration and at the end of the model burn-in period.

Speci-es	Name	Pre-calibration Value (M)	Calibrated Value (M)	Basis for values			Post model burn-in (M)
				Ref	Calc	Calib	
Digestive module							
$x(1)$	Leucine in the stomach	0 or 'leucine input at t_x'*	0	☐	☐	☐	0
$x(2)$	Leucine in the gut	0	0	☐	☐	☐	0
Leucine kinetic module							
$x(4)$	Plasma leucine	1.21×10^{-4} [S1]	1.21×10^{-4}	☒	☐	☐	1.26×10^{-4}
$x(5)$	Plasma KIC	2.58×10^{-5} [S38]	2.58×10^{-5}	☒	☐	☐	1.63×10^{-5}
$x(6)$	Intracellular leucine	1.28×10^{-4} [S4]	1.28×10^{-4}	☒	☐	☒	8.91×10^{-5}
$x(7)$	Intracellular KIC	N/a	1.15×10^{-5}	☐	☐	☒	8.42×10^{-6}
$x(8)$	Protein	1.34×10^{-1} [S39]	1.34×10^{-1}	☒	☒	☐	1.34×10^{-1}
mTOR signalling module							
$x(9)$	IR-β	0.06×10^{-9} – 0.24×10^{-9} [S40]	0.84×10^{-9}	☒	☒	☐	0.80×10^{-9}
$x(10)$	p-IR-β^{Y1164}	N/a**	0.008×10^{-9}	☐	☒	☐	0.05×10^{-9}
$x(11)$	p-IR-$\beta_{refractory}$	N/a**	0.008×10^{-9}	☐	☒	☐	0.01×10^{-9}
$x(12)$	IRS1	0.81×10^{-9} – 3.11×10^{-9} [S41]	0.21×10^{-9}	☒	☒	☐	0.13×10^{-9}
$x(13)$	p-IRS1^{Y}	N/a**	0.002×10^{-9}	☐	☒	☐	0.007×10^{-9}
$x(14)$	p-IRS1^{S}	N/a**	0.002×10^{-9}	☐	☒	☐	0.03×10^{-9}
$x(15)$	PI3K	0.10×10^{-9} – 0.39×10^{-9} [S40]	0.22×10^{-9}	☒	☐	☐	0.19×10^{-9}
$x(16)$	p-IRS1^{Y}/PI3K$_{clx}$	N/a**	0.002×10^{-9}	☐	☒	☐	0.04×10^{-9}
$x(17)$	PDK1	0.71×10^{-9} – 34.3×10^{-9} [S40-41]	0.84×10^{-9}	☒	☐	☐	0.74×10^{-9}
$x(18)$	p-PDK1	N/a**	0.008×10^{-9}	☐	☒	☐	0.11×10^{-9}
$x(19)$	Akt	0.37×10^{-9} – 7.78×10^{-9} [S40-41]	10.0×10^{-9}	☒	☐	☐	9.30×10^{-9}
$x(20)$	p-Akt^{T308}	N/a**	0.10×10^{-9}	☐	☒	☐	0.50×10^{-9}
$x(21)$	p-Akt^{S473}	N/a**	0.10×10^{-9}	☐	☒	☐	0.25×10^{-9}
$x(22)$	p-Akt^{T308,S473}	N/a**	0.10×10^{-9}	☐	☒	☐	0.24×10^{-9}
$x(23)$	TSC$_{clx}$	N/a	0.69×10^{-9}	☒	☒	☐	0.61×10^{-9}
$x(24)$	p-TSC$_{clx}$	N/a**	0.007×10^{-9}	☐	☒	☐	0.08×10^{-9}

$x(25)$	$\text{mTORC1}_{inactive}$	0.23×10^{-9} – 2.50×10^{-9} (MTOR) [S40-41] / 3.14×10^{-9} – 72.1×10^{-9} (RPTOR) [S40-41]	0.30×10^{-9}	☒	☒	☐	0.29×10^{-9}
$x(26)$	mTORC1_{active}	N/a**	0.003×10^{-9}	☐	☒	☐	0.009×10^{-9}
$x(27)$	p70S6K1	3.76×10^{-9} – 14.5×10^{-9} [S40]	8.82×10^{-9}	☒	☒	☐	7.72×10^{-9}
$x(28)$	p-p70S6K1^{T389}	N/a**	0.09×10^{-9}	☐	☒	☐	1.18×10^{-9}
$x(29)$	$\text{PI3K}_{variant}$	N/a	0.21×10^{-9}	☐	☒	☐	0.20×10^{-9}
$x(30)$	$\text{p-PI3K}_{variant}$	N/a	0.002×10^{-9}	☐	☒	☐	0.003×10^{-9}
$x(31)$	mTORC2	1.53×10^{-9} – 5.88×10^{-9} (RICTOR) [S40]	0.43×10^{-9}	☒	☒	☐	0.41×10^{-9}
$x(32)$	p-mTORC2^{S2481}	N/a**	0.004×10^{-9}	☐	☒	☐	0.03×10^{-9}
Insulin oscillatory module[37]							
$x(3)$	Plasma insulin	2.80×10^{-11} [S42]	2.80×10^{-11}	☒	☐	☐	2.72×10^{-11}
$x(35)$	Intercellular insulin	N/a***	0	☐	☐	☐	7.93×10^{-12}
$x(36)$	Plasma glucose	N/a***	0	☐	☐	☐	2.30×10^{-3}
$x(37)$	x1	N/a***	0	☐	☐	☐	4.94×10^{-12}
$x(38)$	x2	N/a***	0	☐	☐	☐	4.87×10^{-12}
$x(39)$	x3	N/a***	0	☐	☐	☐	4.79×10^{-12}

Calc, calculations; Calib, calibrations; Ref, references

*Leucine input at t_x corresponds to the leucine dose and timing that corresponds to the calibration and validation simulation.

**We were unable to locate concentrations of phosphorylated proteins, such that we assumed the initial concentration of the phospho-proteins were 1% of the respective non-phosphorylated protein.

***Sturis et al.[S37] did not provide initial concentrations for the respective species. We set the initial value to zero and allowed the model to achieve a steady-state value during the model burn-in period.

Table S5. Model parameter values. Related to Figure 2.

Model parameter values before and after model calibration.

Parameter name	Original		Pre-calib. Value	Calibrated Value	Units	Basis for value			
	Value	Units				Ref	Calc	Calib	Est^
Digestive module									
k_1	N/a*			5.91×10^{-1}	min^{-1}	☒	☐	☐	☒[S43-45]
k_2	N/a*			1.86×10^{-3}	min^{-1}	☒	☐	☐	☒[S43-45]
k_{68}	N/a*			4.98×10^{-3}	min^{-1}	☒	☐	☐	☒[S43-45]
k_3	N/a*			1.11×10^{-2}	min^{-1}	☒	☐	☐	☒[S43-45]
Blood Plasma									
k_4	N/a#		2.01×10^{-8}	2.29×10^{-8}	min^{-1}	☐	☐	☒	☒
Leucine kinetic module									
k_6	1.61×10^{-7} [S46]	$mol\cdot min^{-1}\cdot 100mL^{-1}$	3.14×10^{-2}	7.34×10^{-2}	min^{-1}	☒	☒	☒	☐
k_7	1.60×10^{-7} [S46]	$mol\cdot min^{-1}\cdot 100mL^{-1}$	6.01×10^{-4}	6.12×10^{-4}	min^{-1}	☒	☒	☒	☐
k_{15}	1.30×10^{-7} [S46]	$mol\cdot min^{-1}\cdot 100mL^{-1}$	5.33×10^{4}	5.29×10^{4}	$mol^{-1}\cdot min^{-1}$	☒	☒	☒	☐
k_9	1.49×10^{-7} [S46]	$mol\cdot min^{-1}\cdot 100mL^{-1}$	5.57×10^{-15}	9.52×10^{-15}	$mol\cdot min^{-1}$	☒	☒	☒	☐
k_{10}	9.53×10^{-8} [S46]	$mol\cdot min^{-1}\cdot 100mL^{-1}$	1.18×10^{-2}	1.17×10^{-2}	min^{-1}	☒	☒	☒	☐
k_{11}	7.35×10^{-8} [S46]	$mol\cdot min^{-1}\cdot 100mL^{-1}$	2.93×10^{-2}	2.87×10^{-2}	min^{-1}	☒	☒	☒	☐
k_{12}	N/a#		5.33×10^{-2}	5.29×10^{-2}	min^{-1}	☒	☐	☒	☒ [S47]
k_{13}	N/a#		1.90×10^{-2}	1.86×10^{-2}	min^{-1}	☒	☐	☒	☒ [S47]
k_{14}	1.96×10^{-8} [S46]	$mol\cdot min^{-1}\cdot 100mL^{-1}$	9.56×10^{-2}	9.53×10^{-2}	min^{-1}	☒	☒	☒	☐
mTOR signalling module									
k_{16}	0.0254 [S48]	A.U.	3.37×10^{7}	3.57×10^{7}	$mol^{-1}\cdot min^{-1}$	☒	☐	☒	☒
k_{17}	0.149 [S48]	A.U.	7.27×10^{-2}	7.25×10^{-2}	min^{-1}	☒	☐	☒	☒
k_{18}	0.0310 [S48]	A.U.	2.93×10^{-1}	2.96×10^{-1}	min^{-1}	☒	☐	☒	☒
k_{19}	0.135 [S48]	A.U.	9.57×10^{6}	9.47×10^{6}	$mol^{-1}\cdot min^{-1}$	☒	☐	☒	☒
k_{20}	3.28×10^{-3} [S48]	A.U.	2.14×10^{-1}	2.22×10^{-1}	min^{-1}	☒	☐	☒	☒

k_{21}	1 [S48]	A.U.	5.78×10^{4}	5.67×10^{4}	$mol^{-1}\cdot min^{-1}$	☒	☐	☒	☒
k_{22}	1.00×10^{-4} [S48]	A.U.	6.88×10^{-3}	6.90×10^{-3}	min^{-1}	☒	☐	☒	☒
k_{23}	7.06×10^{12} [S49]	min^{-1}	1.65×10^{8}	2.09×10^{8}	$mol^{-1}\cdot min^{-1}$	☒	☒	☒	☒
k_{24}	10 [S49]	min^{-1}	1.97×10^{-1}	1.92×10^{-1}	min^{-1}	☒	☒	☒	☒
k_{25}	N/a#		5.86×10^{7}	5.84×10^{7}	$mol^{-1}\cdot min^{-1}$	☐	☐	☒	☒
k_{26}	N/a#		4.05×10^{-1}	4.10×10^{-1}	min^{-1}	☐	☐	☒	☒
k_{27}	0.700 [S48]	A.U.	9.05×10^{8}	9.08×10^{8}	$mol^{-1}\cdot min^{-1}$	☒	☐	☒	☒
k_{28}	4.07 [S48]	A.U.	5.03×10^{1}	5.05×10^{1}	min^{-1}	☒	☐	☒	☒
k_{29}	N/a#		5.46×10^{8}	5.50×10^{8}	$mol^{-1}\cdot min^{-1}$	☐	☐	☒	☒
k_{30}	N/a#		1.03×10^{1}	9.98	min^{-1}	☐	☐	☒	☒
k_{31}	4.51 [S48]	A.U.	9.28×10^{8}	9.28×10^{8}	$mol^{-1}\cdot min^{-1}$	☒	☐	☒	☒
k_{32}	7.53 [S48]	A.U.	6.28	6.31	min^{-1}	☒	☐	☒	☒
k_{33}	N/a#		3.67×10^{9}	3.82×10^{9}	$mol^{-1}\cdot min^{-1}$	☐	☐	☒	☒
k_{34}	N/a#		6.47	6.47	min^{-1}	☐	☐	☒	☒
k_{35}	1.00×10^{-4} [S48]	A.U.	2.13×10^{4}	2.09×10^{4}	$mol^{-1}\cdot min^{-1}$	☒	☐	☒	☒
k_{36}	6.27×10^{-3} [S48]	A.U.	9.31×10^{5}	9.23×10^{5}	$mol^{-1}\cdot min^{-1}$	☒	☐	☒	☒
k_{37}	8.13×10^{-3} [S48]	A.U.	8.47×10^{-2}	8.54×10^{-2}	min^{-1}	☒	☐	☒	☒
k_{38}	1.00 [S48]	A.U.	2.04×10^{5}	1.98×10^{5}	$mol^{-1}\cdot min^{-1}$	☒	☐	☒	☒
k_{39}	0.0514 [S48]	A.U.	3.36×10^{-1}	3.49×10^{-1}	$mol^{-1}\cdot min^{-1}$	☒	☐	☒	☒
k_{40}	5.74×10^{-3} [S48]	A.U.	4.44×10^{7}	4.70×10^{7}	$mol^{-1}\cdot min^{-1}$	☒	☐	☒	☒
k_{41}	N/a#		8.14×10^{4}	8.16×10^{4}	$mol^{-1}\cdot min^{-1}$	☐	☐	☒	☒
k_{42}	5.28×10^{-3} [S48]	A.U.	7.18×10^{-2}	7.34×10^{-2}	min^{-1}	☒	☐	☒	☒
k_{43}	N/a#		7.59×10^{5}	7.72×10^{5}	$mol^{-1}\cdot min^{-1}$	☐	☐	☒	☒
k_{44}	1.00 [S48]	A.U.	1.64×10^{6}	2.10×10^{6}	$mol^{-1}\cdot min^{-1}$	☒	☐	☒	☒
k_{45}	2.32×10^{-4} [S48]	A.U.	1.02×10^{-1}	1.63×10^{-1}	min^{-1}	☒	☐	☒	☒
k_{46}	3.19×10^{-2} [S48]	A.U.	2.23×10^{7}	2.26×10^{7}	$mol^{-1}\cdot min^{-1}$	☒	☐	☒	☒
k_{47}	2.56×10^{-2} [S48]	A.U.	2.90×10^{-2}	2.95×10^{-2}	min^{-1}	☒	☐	☒	☒
k_{69}	N/a#		4.40×10^{-17}	4.41×10^{-17}	$mol\cdot min^{-1}$	☒	☐	☒	☒ [S50]

Insulin oscillatory module [S37]									
k_{48} (V_p)	3	L	3	3	L	☒	☐	☐	☐
k_{49} (V_i)	11	L	11	11	L	☒	☐	☐	☐
k_{50} (V_g)	10	L	10	10	L	☒	☐	☐	☐
k_{51} (E)	0.2	L·min^{-1}	0.2	9.55×10^{-2}	L·min^{-1}	☒	☐	☒	☐
k_{52} (t_p)	6	min	6	3.62	min	☒	☐	☒	☐
k_{53} (t_i)	100	min	100	92.6	min	☒	☐	☒	☐
k_{54} (t_d)	36	min	36	28	min	☒	☐	☒	☐
k_{55} (R_m)	210	mU·min^{-1}	1.40×10^{-9}	1.12×10^{-9}	mol·min^{-1}	☒	☒	☒	☐
k_{56} (a_1)	300	mg·L^{-1}	1.67×10^{-3}	9.21×10^{-4}	mol·L^{-1}	☒	☒	☒	☐
k_{57} (C_1)	2000	mg·L^{-1}	1.11×10^{-2}	9.61×10^{-3}	mol·L^{-1}	☒	☒	☒	☐
k_{58} (U_b)	72	mg·min^{-1}	4.00×10^{-4}	2.70×10^{-4}	mol·min^{-1}	☒	☒	☒	☐
k_{59} (C_2)	144	mg·L^{-1}	7.99×10^{-4}	6.04×10^{-4}	mol·L^{-1}	☒	☒	☒	☐
k_{60} (C_3)	1000	mg·L^{-1}	5.55×10^{-3}	2.62×10^{-3}	mol·L^{-1}	☒	☒	☒	☐
k_{61} (U_0)	40	mg·min^{-1}	2.22×10^{-4}	1.71×10^{-4}	mol·min^{-1}	☒	☒	☒	☐
k_{62} (U_m)	940	mg·min^{-1}	5.22×10^{-3}	4.61×10^{-3}	mol·min^{-1}	☒	☒	☒	☐
k_{63} (β)	1.77	unitless	1.77	1.33	unitless	☒	☐	☒	☐
k_{64} (C_4)	80	mU·L^{-1}	5.34×10^{-10}	3.89×10^{-10}	mol·L^{-1}	☒	☒	☒	☐
k_{65} (R_g)	180	mg·min^{-1}	9.99×10^{-4}	7.94×10^{-4}	mol·min^{-1}	☒	☒	☒	☐
k_{66} (α)	0.29	L·mU^{-1}	4.35×10^{10}	4.50×10^{10}	L·mol^{-1}	☒	☒	☒	☐
k_{67} (C_5)	26	mU·L^{-1}	1.73×10^{-10}	1.39×10^{-10}	mol·L^{-1}	☒	☒	☒	☐

A.U., arbitrary units; Calc, calculations; Calib, calibrations; Est, estimations; N/a, not applicable; Ref, references

^Citations specific to the estimation column were used for the estimation of the respective parameter.

*We manually derived the parameter values for the digestive system module using the plasma leucine time-course[S1] and two published values. 1) True ileal digestibility[S45]: approximately 90% of the ingested leucine is absorbed into the blood stream (i.e., 10% of the ingested leucine bolus is excreted). 2) First-pass splanchnic extraction[S43-44]: in young adults, the first-pass splanchnic extraction of amino acids is 23-29%.

#Parameter values that have an original value of 'N/a' were added during the model topology expansion. These parameters did not have references to directly infer the parameter value, such that we used a heuristic approach to derive the pre-calibration values. Our heuristic approach required each parameter value to produce a reasonable qualitative kinetic for the respective model species. A reasonable qualitative kinetic for the model species was defined as: 1) does not remain at zero throughout the simulation, 2) is not fully saturated throughout the model simulation, and 3) has

a reasonable dynamic following model simulation (i.e., there was a realistic increase and decrease in the species that corroborated with upstream species).

Table S6. Calibration dataset characteristics. Related to Figure 2A.

Species	Study	Subjects (M, F)	Age (SEM)	Intervention	Leucine content	EAA content	Whey protein
Plasma leucine	Glynn et al.[S1]	7 (3, 4)	29 (2)	A single bolus provided (noncaloric, noncaffeinated carbonated beverage, 500 mL)	3.5 g	10.0 g	0 g
$F_{m,a}$							
$F_{m,0}$							
FSR							
Plasma insulin							
Intracellular leucine	Drummond et al.[S4]	7 (3, 4)	29.0 (2.0)	A single bolus provided (in a 500 mL solution)	3.5 g	10.0 g	0 g
p-Akt^{S473}	Meta-analyzed						
p-$p70S6K^{T389}$	Meta-analyzed						

Table S7. Validation dataset characteristics. Related to Figure 2B,C.

Study	Subjects (M, F)	Age (SEM)	Intervention	Leucine content	EAA content	Whey protein
Glynn et al.[S1]	7 (3, 4)	32 (2)	Single bolus provided (noncaloric, noncaffeinated carbonated beverage, 500 mL)	1.85 g	10.0 g	0 g
Mitchell et al.[S2]	8 (8, 0)	19.7 (0.5)	Single bolus provided (in aqueous solution, 250 mL)	3.59 g	15.0 g	0 g
Mitchell et al.[S2]	8 (8, 0)	21.5 (1.1)	Four equal fractions were ingested at 45-min intervals (in aqueous solution, 250 mL)	3.59 g (4 x 0.9 g)	15.0 g	0 g
Drummond et al.[S4]	7 (3, 4)	29.0 (2.0)	Single bolus provided (in a 500 mL solution)	3.5 g	10.0 g	0 g
Wilkinson et al.[S3]	7 (7,0)	21 (0.3)	Leucine intervention: Single bolus provided (with ~400 mL of water)	3.42 g	3.42 g	0 g
Dickinson et al.[S5]	8 (3,5)	25 (2)	A 10-gram EAA solution was ingested.	1.8 g	10 g	0 g

Table S8. Amino acid profile for each intervention. Related to Figure 2.

Dataset	Leu (g)	His (g)	Iso (g)	Lys (g)	Met (g)	Phe (g)	Thr (g)	Trp (g)	Val (g)	Total AA (g)
Glynn et al.[S1] – leucine	3.50	0.80	0.80	1.20	0.30	1.40	1.00	0	1.00	10.00
Glynn et al.[S1] – control	1.85	1.10	1.00	1.55	0.30	1.55	1.45	0	1.20	10.00
Mitchell et al.[S2] – bolus	3.59	1.21	1.73	3.07	0.95	0.91	0.48	1.13	1.86	14.93
Mitchell et al.[S2] – pulsatile	3.59	1.21	1.73	3.07	0.95	0.91	0.48	1.13	1.86	14.93
Drummond et al.[S4]	3.50	0.80	0.80	1.20	0.30	1.40	1.00	0	1.00	10.00
Dickinson et al.[S5]	1.80	1.10	1.00	1.60	0.30	1.60	1.40	0	1.20	10.00
Wilkinson et al.[S3]	3.42	0	0	0	0	0	0	0	0	3.42

*Atherton et al.[S47], Moore et al.[S7], and Mazzulla et al.[S8] are not included in this table as each study provided participants with whey protein and did not report the amino acid profile of the intervention.

Supplemental References

S1. Glynn, E.L., Fry, C.S., Drummond, M.J., Timmerman, K.L., Dhanani, S., Volpi, E., and Rasmussen, B.B. (2010). Excess leucine intake enhances muscle anabolic signaling but not net protein anabolism in young men and women. The Journal of nutrition *140*, 1970–1976. 10.3945/jn.110.127647.

S2. Mitchell, W.K., Phillips, B.E., Williams, J.P., Rankin, D., Lund, J.N., Smith, K., and Atherton, P.J. (2015). A dose- rather than delivery profile-dependent mechanism regulates the "muscle-full" effect in response to oral essential amino acid intake in young men. The Journal of nutrition *145*, 207–214. 10.3945/jn.114.199604.

S3. Wilkinson, D.J., Hossain, T., Hill, D.S., Phillips, B.E., Crossland, H., Williams, J., Loughna, P., Churchward-Venne, T.A., Breen, L., Phillips, S.M., et al. (2013). Effects of leucine and its metabolite beta-hydroxy-beta-methylbutyrate on human skeletal muscle protein metabolism. The Journal of physiology *591*, 2911–2923. 10.1113/jphysiol.2013.253203.

S4. Drummond, M.J., Glynn, E.L., Fry, C.S., Timmerman, K.L., Volpi, E., and Rasmussen, B.B. (2010). An increase in essential amino acid availability upregulates amino acid transporter expression in human skeletal muscle. American journal of physiology. Endocrinology and metabolism *298*, E1011-8. 10.1152/ajpendo.00690.2009.

S5. Dickinson, J.M., Fry, C.S., Drummond, M.J., Gundermann, D.M., Walker, D.K., Glynn, E.L., Timmerman, K.L., Dhanani, S., Volpi, E., and Rasmussen, B.B. (2011). Mammalian target of rapamycin complex 1 activation is required for the stimulation of human skeletal muscle protein synthesis by essential amino acids. The Journal of nutrition *141*, 856–862. 10.3945/jn.111.139485.

S6. Atherton, P.J., Etheridge, T., Watt, P.W., Wilkinson, D., Selby, A., Rankin, D., Smith, K., and Rennie, M.J. (2010). Muscle full effect after oral protein: time-dependent concordance and discordance between human muscle protein synthesis and mTORC1 signaling. The American journal of clinical nutrition *92*, 1080–1088. 10.3945/ajcn.2010.29819.

S7. Moore, D.R., Robinson, M.J., Fry, J.L., Tang, J.E., Glover, E.I., Wilkinson, S.B., Prior, T., Tarnopolsky, M.A., and Phillips, S.M. (2009). Ingested protein dose response of muscle and albumin protein synthesis after resistance exercise in young men. The American journal of clinical nutrition *89*, 161–168. 10.3945/ajcn.2008.26401.

S8. Mazzulla, M., Parel, J.T., Beals, J.W., VAN Vliet, S., Abou Sawan, S., West, D.W.D., Paluska, S.A., Ulanov, A.V., Moore, D.R., and Burd, N.A. (2017). Endurance Exercise Attenuates Postprandial Whole-Body Leucine Balance in Trained Men. Med Sci Sports Exerc *49*, 2585–2592. 10.1249/MSS.0000000000001394.

S9. Alessi, D.R., James, S.R., Downes, C.P., Holmes, A.B., Gaffney, P.R., Reese, C.B., and Cohen, P. (1997). Characterization of a 3-phosphoinositide-dependent protein kinase which phosphorylates and activates protein kinase Balpha. Curr Biol *7*, 261–269. 10.1016/s0960-9822(06)00122-9.

S10. Bertuzzi, A., Conte, F., Mingrone, G., Papa, F., Salinari, S., and Sinisgalli, C. (2016). Insulin Signaling in Insulin Resistance States and Cancer: A Modeling Analysis. PloS one *11*, e0154415. 10.1371/journal.pone.0154415.

S11. Cybulski, N., and Hall, M.N. (2009). TOR complex 2: a signaling pathway of its own. Trends Biochem Sci *34*, 620–627. 10.1016/j.tibs.2009.09.004.

S12. Dalle Pezze, P., Sonntag, A.G., Thien, A., Prentzell, M.T., Gödel, M., Fischer, S., Neumann-Haefelin, E., Huber, T.B., Baumeister, R., Shanley, D.P., et al. (2012). A dynamic network model of mTOR signaling reveals TSC-independent mTORC2 regulation. Science Signaling *5*. 10.1126/scisignal.2002469.

S13. Jeyapalan, A.S., Orellana, R.A., Suryawan, A., O'Connor, P.M.J., Nguyen, H.V., Escobar, J., Frank, J.W., and Davis, T.A. (2007). Glucose stimulates protein synthesis in skeletal muscle of neonatal pigs through an AMPK- and mTOR-independent process. Am J Physiol Endocrinol Metab *293*, E595-603. 10.1152/ajpendo.00121.2007.

S14. Cusi, K., Maezono, K., Osman, A., Pendergrass, M., Patti, M.E., Pratipanawatr, T., DeFronzo, R.A., Kahn, C.R., and Mandarino, L.J. (2000). Insulin resistance differentially affects the PI 3-kinase- and MAP kinase-mediated signaling in human muscle. J Clin Invest *105*, 311–320. 10.1172/JCI7535.

S15. Bouzakri, K., Roques, M., Gual, P., Espinosa, S., Guebre-Egziabher, F., Riou, J.-P., Laville, M., Le Marchand-Brustel, Y., Tanti, J.-F., and Vidal, H. (2003). Reduced activation of phosphatidylinositol-3 kinase and increased serine 636 phosphorylation of insulin receptor substrate-1 in primary culture of skeletal muscle cells from patients with type 2 diabetes. Diabetes *52*, 1319–1325. 10.2337/diabetes.52.6.1319.

S16. Dibble, C.C., Asara, J.M., and Manning, B.D. (2009). Characterization of Rictor phosphorylation sites reveals direct regulation of mTOR complex 2 by S6K1. Molecular and cellular biology *29*, 5657–5670. 10.1128/MCB.00735-09.

S17. Magnuson, B., Ekim, B., and Fingar, D.C. (2012). Regulation and function of ribosomal protein S6 kinase (S6K) within mTOR signalling networks. The Biochemical journal *441*, 1–21. 10.1042/BJ20110892.

S18. Yi, Z., Langlais, P., De Filippis, E.A., Luo, M., Flynn, C.R., Schroeder, S., Weintraub, S.T., Mapes, R., and Mandarino, L.J. (2007). Global assessment of regulation of phosphorylation of insulin receptor substrate-1 by insulin in vivo in human muscle. Diabetes *56*, 1508–1516. 10.2337/db06-1355.

S19. Buel, G.R., and Blenis, J. (2016). Seeing mTORC1 specificity. Science (New York, N.Y.) *351*, 25–26. 10.1126/science.aad9696.

S20. Chiang, G.G., and Abraham, R.T. (2005). Phosphorylation of mammalian target of rapamycin (mTOR) at Ser-2448 is mediated by p70S6 kinase. The Journal of biological chemistry *280*, 25485–25490. 10.1074/jbc.M501707200.

S21. Dibble, C.C., and Cantley, L.C. (2015). Regulation of mTORC1 by PI3K signaling. Trends in cell biology *25*, 545–555. 10.1016/j.tcb.2015.06.002.

S22. Figueiredo, V.C., Markworth, J.F., and Cameron-Smith, D. (2017). Considerations on mTOR regulation at serine 2448: implications for muscle metabolism studies. Cellular and molecular life sciences : CMLS *74*, 2537–2545. 10.1007/s00018-017-2481-5.

S23. Holz, M.K., and Blenis, J. (2005). Identification of S6 kinase 1 as a novel mammalian target of rapamycin (mTOR)-phosphorylating kinase. The Journal of biological chemistry *280*, 26089–26093. 10.1074/jbc.M504045200.

S24. Sancak, Y., Bar-Peled, L., Zoncu, R., Markhard, A.L., Nada, S., and Sabatini, D.M. (2010). Ragulator-Rag complex targets mTORC1 to the lysosomal surface and is necessary for its activation by amino acids. Cell *141*, 290–303. 10.1016/j.cell.2010.02.024.

S25. Saxton, R.A., Knockenhauer, K.E., Wolfson, R.L., Chantranupong, L., Pacold, M.E., Wang, T., Schwartz, T.U., and Sabatini, D.M. (2016). Structural basis for leucine sensing by the Sestrin2-mTORC1 pathway. Science (New York, N.Y.) *351*, 53–58. 10.1126/science.aad2087.

S26. Zoncu, R., Sabatini, D.M., and Efeyan, A. (2011). mTOR: from growth signal integration to cancer, diabetes and ageing. Nat Rev Mol Cell Biol *12*, 21–35. 10.1038/nrm3025.

S27. Fonseca, B.D., Smith, E.M., Yelle, N., Alain, T., Bushell, M., and Pause, A. (2014). The ever-evolving role of mTOR in translation. Seminars in cell & developmental biology *36*, 102–112. 10.1016/j.semcdb.2014.09.014.

S28. Pearce, L.R., Komander, D., and Alessi, D.R. (2010). The nuts and bolts of AGC protein kinases. Nat Rev Mol Cell Biol *11*, 9–22. 10.1038/nrm2822.

S29. Song, Y.-H., Godard, M., Li, Y., Richmond, S.R., Rosenthal, N., and Delafontaine, P. (2005). Insulin-like growth factor I-mediated skeletal muscle hypertrophy is characterized by increased mTOR-p70S6K signaling without increased Akt phosphorylation. J Investig Med *53*, 135–142. 10.2310/6650.2005.00309.

S30. Kuramoto, N., Nomura, K., Kohno, D., Kitamura, T., Karsenty, G., Hosooka, T., and Ogawa, W. (2021). Role of PDK1 in skeletal muscle hypertrophy induced by mechanical load. Sci Rep *11*, 3447. 10.1038/s41598-021-83098-z.

S31. Huang, J., and Manning, B.D. (2009). A complex interplay between Akt, TSC2 and the two mTOR complexes. Biochem Soc Trans *37*, 217–222. 10.1042/BST0370217.

S32. Alessi, D.R., Andjelkovic, M., Caudwell, B., Cron, P., Morrice, N., Cohen, P., and Hemmings, B.A. (1996). Mechanism of activation of protein kinase B by insulin and IGF-1. EMBO J *15*, 6541–6551.

S33. Sedaghat, A.R., Sherman, A., and Quon, M.J. (2002). A mathematical model of metabolic insulin signaling pathways. American journal of physiology. Endocrinology and metabolism *283*, E1084-101. 10.1152/ajpendo.00571.2001.

S34. Manning, B.D., Tee, A.R., Logsdon, M.N., Blenis, J., and Cantley, L.C. (2002). Identification of the tuberous sclerosis complex-2 tumor suppressor gene product tuberin as a target of the phosphoinositide 3-kinase/akt pathway. Mol Cell *10*, 151–162. 10.1016/s1097-2765(02)00568-3.

S35. Miyazaki, M., McCarthy, J.J., Fedele, M.J., and Esser, K. a (2011). Early activation of mTORC1 signalling in response to mechanical overload is independent of phosphoinositide 3-kinase/Akt signalling. The Journal of physiology *589*, 1831–1846. 10.1113/jphysiol.2011.205658.

S36. Tolic, I.M., Mosekilde, E., and Sturis, J. (2000). Modeling the insulin-glucose feedback system: the significance of pulsatile insulin secretion. Journal of theoretical biology *207*, 361–375. 10.1006/jtbi.2000.2180.

S37. Sturis, J., Polonsky, K.S., Mosekilde, E., and Van Cauter, E. (1991). Computer model for mechanisms underlying ultradian oscillations of insulin and glucose. The American journal of physiology *260*, E801-9. 10.1152/ajpendo.1991.260.5.E801.

S38. Tessari, P., Inchiostro, S., Zanetti, M., and Barazzoni, R. (1995). A model of skeletal muscle leucine kinetics measured across the human forearm. The American journal of physiology *269*, E127-36.

S39. Wolfe, R.R., Park, S., Kim, I.-Y., Starck, C., Marquis, B.J., Ferrando, A.A., and Moughan, P.J. (2019). Quantifying the contribution of dietary protein to whole body protein kinetics: examination of the intrinsically labeled proteins method. American journal of physiology. Endocrinology and metabolism *317*, E74–E84. 10.1152/ajpendo.00294.2018.

S40. Wiśniewski, J.R., Hein, M.Y., Cox, J., and Mann, M. (2014). A "proteomic ruler" for protein copy number and concentration estimation without spike-in standards. Mol Cell Proteomics *13*, 3497–3506. 10.1074/mcp.M113.037309.

S41. Gonzalez-Franquesa, A., Stocks, B., Chubanava, S., Hattel, H.B., Moreno-Justicia, R., Peijs, L., Treebak, J.T., Zierath, J.R., and Deshmukh, A.S. (2021). Mass-spectrometry-based proteomics reveals mitochondrial supercomplexome plasticity. Cell Rep *35*, 109180. 10.1016/j.celrep.2021.109180.

S42. Reidy, P.T., Walker, D.K., Dickinson, J.M., Gundermann, D.M., Drummond, M.J., Timmerman, K.L., Fry, C.S., Borack, M.S., Cope, M.B., Mukherjea, R., et al. (2013). Protein blend ingestion following resistance exercise promotes human muscle protein synthesis. The Journal of nutrition *143*, 410–416. 10.3945/jn.112.168021.

S43. Boirie, Y., Gachon, P., and Beaufrère, B. (1997). Splanchnic and whole-body leucine kinetics in young and elderly men. The American journal of clinical nutrition *65*, 489–495. 10.1093/ajcn/65.2.489.

S44. Volpi, E., Mittendorfer, B., Wolf, S.E., and Wolfe, R.R. (1999). Oral amino acids stimulate muscle protein anabolism in the elderly despite higher first-pass splanchnic extraction. The American journal of physiology *277*, E513-20. 10.1152/ajpendo.1999.277.3.E513.

S45. Wolfe, R.R., Park, S., Kim, I.-Y., Starck, C., Marquis, B.J., Ferrando, A.A., and Moughan, P.J. (2019). Quantifying the contribution of dietary protein to whole body protein kinetics: examination of the intrinsically labeled proteins method. American journal of physiology. Endocrinology and metabolism *317*, E74–E84. 10.1152/ajpendo.00294.2018.

S46. Tessari, P., Inchiostro, S., Zanetti, M., and Barazzoni, R. (1995). A model of skeletal muscle leucine kinetics measured across the human forearm. The American journal of physiology *269*, E127-36.

S47. Atherton, P.J., Etheridge, T., Watt, P.W., Wilkinson, D., Selby, A., Rankin, D., Smith, K., and Rennie, M.J. (2010). Muscle full effect after oral protein: time-dependent concordance and discordance

between human muscle protein synthesis and mTORC1 signaling. The American journal of clinical nutrition *92*, 1080–1088. 10.3945/ajcn.2010.29819.

S48. Dalle Pezze, P., Sonntag, A.G., Thien, A., Prentzell, M.T., Gödel, M., Fischer, S., Neumann-Haefelin, E., Huber, T.B., Baumeister, R., Shanley, D.P., et al. (2012). A dynamic network model of mTOR signaling reveals TSC-independent mTORC2 regulation. Science Signaling *5*. 10.1126/scisignal.2002469.

S49. Chew, Y.H., Shia, Y.L., Lee, C.T., Majid, F.A.A., Chua, L.S., Sarmidi, M.R., and Aziz, R.A. (2009). Modeling of glucose regulation and insulin-signaling pathways. Molecular and Cellular Endocrinology *303*, 13–24. 10.1016/j.mce.2009.01.018.

S50. Neel, B.A., Lin, Y., and Pessin, J.E. (2013). Skeletal muscle autophagy: a new metabolic regulator. Trends Endocrinol Metab *24*, 635–643. 10.1016/j.tem.2013.09.004.
